# Parameter optimization of the spiral fertiliser discharger for mango orchards based on the discrete element method and genetic algorithm

**DOI:** 10.3389/fpls.2023.1169091

**Published:** 2023-11-06

**Authors:** Liang Zhao, Hongping Zhou, Linyun Xu, Weidong Yuan, Minghong Shi, Jian Zhang, Zhong Xue

**Affiliations:** ^1^ College of Mechatronics Engineering, Nanjing Forestry University, Nanjing, China; ^2^ South Subtropical Crops Research Institute, Chinese Academy of Tropical Agricultural Sciences, Zhanjiang, China

**Keywords:** mango orchard, spiral fertiliser discharger, parameter optimisation, genetic algorithm, discrete element method, response surface methodology

## Abstract

**Introduction:**

In order to solve the problems of inaccurate fertilization, unstable fertilization and low fertiliserutilization rate in mango orchard.

**Methods:**

A small spiral fertiliser discharger was designed based on the agronomic characteristics of fertilization in mango orchard. The fertilizing performance test and parameter optimization of thespiral fertiliser discharger were carried out by combining bench and simulation test. Firstly, the main influencing factors of the fertilizing performance of the spiral fertiliser discharger were analyzed by theoretical calculation formula, and the range of its value was preliminarily determined. At the same time, the digital and discrete element models of the spiral fertiliser discharger were established. Then,the discrete element model of granular fertiliser was established on the basis of the physical and related mechanical simulation parameters of granular fertiliser obtained by experimental statistics.Taking the variable coefficient of fertilizing stability as the response value, the method of singlefactor simulation fertilizing test was used to explore the parameters that have a significant influence on the variable coefficient of fertilizing stability. The response surface method (RSM) was used tosimulate the fertilizing performance of three significant parameters. Based on the quadraticregression orthogonal rotation combination design test, a second-order regression mathematicalmodel between the variable coefficient of fertilizing stability and the significant parameters wasestablished. The variable coefficient of fertilizing stability was as small as possible. The geneticalgorithm (GA) was used to optimize the regression model. Finally, the verification test of thefluidity and applicability of different fertilisers was carried out.

**Results:**

The results of single factor test showed that the diameter of spiral blade, pitch and rotationalspeed of fertilizing shaft have significant influence on the variable coefficient of fertilizing stability.The optimal parameter combination of the spiral fertiliser discharger was obtained: 98.44 mm for thediameter of spiral blade, 54.8 mm for the pitch, and 24.43 r/min for the rotational speed of fertilizingshaft. The verification results showed that the average relative error of the test was small, and themass flow rate of different fertilisers and the variable coefficient of fertilizing stability could meetthe agronomic requirements of fertilization in mango orchards. The reliability of the discrete elementsimulation test results and research methods of the spiral fertiliser discharger was verified.

**Conclusion:**

The results and methods of this study can provide reference for the development of mangoorchard fertilization machinery and related fertilizing performance test.

## Highlights

1. DEM-GA is used to optimize the parameters of spiral fertilizer discharger.
2. The optimal combination of parameters is obtained by optimizing regression model.
3. Fertilizing stability of spiral fertiliser discharger for mango orchard is improved.
4. The fertilizer utilization rate and environmental protection effect were improved.
5. Providing references for optimised design of mango orchard fertilization machinery.
6. Providing references for related study on fertilizing performance in other orchards.

## 1 Introduction

Mango is one of the world’s five major tropical fruits (banana, mango, pineapple, longan, and sugar apple), which has a rich nutrition and unique taste, and it has been favored by people, known as the ‘king of tropical fruits’ (Wei and Aiping, 2021). Mango is native to the Himalayas, India, Bangladesh, Indochina Peninsula, and Malay Peninsula, and in China, it is mainly distributed in Yunnan, Guangxi, Guangdong, Sichuan, Fujian, Guizhou, Taiwan, and Hainan (He et al., 2018). According to statistics, by 2020, the national mango planting area has reached 5.241 million mu. The total yield in China ranks third in the global mango-producing countries (Wei, 2021), and the planting area and yield maintain a steady growth trend.

Fertilization is a crucial operation in mango orchard management, which plays a key role in mango quality and yield (Liu et al., 2021). At present, there are some problems with fertilization in mango orchards in China, such as high intensity, low efficiency, and poor quality of artificial fertilization. Unreasonable fertilization can easily lead to soil compaction, unbalanced nutrition of fruit trees, low fertilizer utilization, and environmental pollution (Huang et al., 2018; Qu et al., 2021). Mechanical fertilization can effectively reduce the labor intensity of fruit farmers, improve fertilization efficiency and quality, and reduce the cost of agricultural production. The fertiliser discharger is a key component of fertilization machinery, and its working performance directly affects fertilization quality (Zhao et al., 2022). Currently, the fertiliser dischargers at home and abroad can be mainly divided into the outer groove wheel type, the spiral type, the centrifugal type, and the rotary disc type. These fertiliser dischargers have their advantages and disadvantages and different applicable scenarios, but there is a certain pulsation degree in the fertiliser discharge operation (Zhu et al., 2018; Liu et al., 2020; Dun et al., 2022), resulting in poor stability and uniformity of fertilization. Therefore, it is necessary to perform optimization and experimental study of fertiliser dischargers to improve fertilization performance.

The traditional test method has been adopted to optimize the fertilizing performance of the fertiliser discharger with low efficiency, high cost, and long cycle, and the simulation test method can effectively solve the above problems. The discrete Element Method (DEM) is a numerical simulation method based on the discontinuity assumption proposed by Professor CUNDALL (Wei and Gao, 2021) in 1971. It can be used to simulate and analyse the interaction between agricultural granular materials and mechanical equipment. It provides a new approach for the digital design of modern agricultural equipment, greatly improves the research and development efficiency of agricultural equipment, and has a good application prospect in agricultural engineering (Liao et al., 2023; Tan et al., 2022; Liu et al., 2022).

In recent years, domestic and foreign scholars have mainly studied the performance of large-scale fertilization for fruits and crops such as apples, bananas, corn, and soybeans based on the discrete element method (DEM). For example, Yuan et al. (2021) investigated the effect of the blade configuration of the auger on the mixing performance of orchard soil-fertiliser particles by the discrete element method and experimental measurement method, and the optimal parameters of blade rotation speed, lateral angle, pitch angle, and blade number were obtained. Liu et al. (Liu et al., 2020). designed a fertilization shunt plate according to the fertilization characteristics of alfalfa, optimised its structural parameters by discrete element simulation test, determined the optimal knob width, tilt angle, and horizontal distance, and verified the accuracy of the simulation results through field experiments. Sun et al. (2020) designed and developed a groove wheel fertilization device by using 3D printing technology. Then, the EDEM simulation test was used to analyse the influence of various factors on the fertilization performance, and the optimal parameters of groove wheel radius, helix angle, rotation speed, and inclination angle were determined. Thaper R. (Thaper, 2014) applied the discrete element method to analyse the relationship between different fertiliser types and leaf shapes and the uniformity of the double disc spreader. By establishing a discrete element simulation model of an orchard centrifugal fertiliser applicator, COETZEE (Coetzee and Lombard, 2011) investigated the influence of structural parameters such as orifice flow and blade inclination angle on the consistency of the orchard centrifugal fertiliser spreader. The experimental results indicated that the discrete element simulation model has a good prediction effect. By using the discrete element method, van Liedekerke et al. (Van Liedekerke et al., 2009). studied the effect of different disc rotating speeds on the performance of a centrifugal seeder. Zhu et al. (2022) designed a self-propelled orchard organic fertiliser strip paver according to the planting status of dwarf and dense apple orchards in China and the agronomic requirements of organic fertiliser strip furrow fertilization. The discrete element method was employed to optimize the structure of the fertiliser discharge port of the strip paver. The optimal structure of the fertiliser discharge port was determined to be an oblique mouth shape, and its fertilization performance was verified by a strip paving test and field test. Song et al. (2020) designed a rotary variable fertiliser discharger according to the distribution pattern of banana roots, and they optimized the parameters of the fertiliser discharger through discrete element simulation analysis. The optimal parameters of the forward speed, the rotation period, the central angle, and the opening size of the curved groove were obtained, and the performance of the fertiliser discharger was verified by prototype and field experiments. Jia et al. (2022) designed a pneumatic centralized precision mixed fertilization device according to the requirement of topdressing in the middle and late stages of maize growth. Based on fluid dynamics and the discrete element coupling method, the optimal inclination angle of the fertiliser outlet, the conveying gas velocity, and the bellows length were obtained, and the fertilization performance was verified by field experiment. Dun et al (Dun et al., 2018). developed a ratio control and position stratified fertilization device that is suitable for the soybean planting pattern in Northeast China, and the ditching fertilization performance of the device was investigated by combining discrete element simulation and field experiment. Liu et al. (Liu et al., 2021). designed a layered quantitative fertilization device according to the rape compartment noodle planting mode and the rape root growth law. The discrete element simulation test was conducted to determine the optimal parameters of the diameter of the retaining rod, the number of the retaining rod groups, and the spacing of the retaining rod groups. Then, the operating performance was verified by bench and field experiments. For the research of optimization methods, Thawkar et al. (Thawkar et al., 2021). proposed a hybrid feature selection method based on butterfly optimization algorithm (BOA) and ant lion optimization algorithm (ALO). The results show that BOAALO is superior to the original BOA and ALO in terms of accuracy, sensitivity, specificity, kappa value, type I and type II errors, and the receiver operating characteristic curve, which can be better applied to the diagnosis of breast cancer. Sayed et al (Sayed et al., 2021). proposed a new hybrid version of convolutional neural network architecture and bald eagle search (BES) optimization. The BES algorithm is used to find the optimal value of the hyperparameters of the SqueezeNet architecture. The overall accuracy of the proposed melanoma skin cancer prediction model is 98.37%, indicating that the method can be better applied to skin lesion classification. Xing et al. (Xing et al., 2023). proposed an improved whale optimization algorithm (QGBWOA). A comparative experiment with dimensions of 10, 30, 50, and 100 was conducted on CEC 2014, and a comparative experiment with dimensions of 30 was conducted on CEC 2020. The experimental results show that the convergence accuracy and convergence speed are significantly improved. Pir et al. (Piri and Mohapatra, 2021). used a multi-objective quadratic binary HHO (MOQBHHO) technique with K-Nearest Neighbor (KNN) method as the wrapper classifier to extract the optimal feature subset, and carried out relevant comparative experiments. The results show that the MOQBHHO proposed in this paper effectively finds a set of non-dominated feature subsets, which has advantages in obtaining the best trade-off between the two fitness evaluation criteria. Santana et al. (2018) used genetic algorithm to optimize the parameters of vacuum cooling treatment conditions for broccoli after harvest. The results showed that GA had good performance for the optimization of broccoli cooling process, and the best conditions for vacuum cooling process of broccoli were obtained. The above research on optimization methods has certain guiding significance for this study.

Though many studies have been conducted on the fertilizing performance of fruits and crops at home and abroad, there are few reports on the fertilizing performance of fertilization machinery in mango orchards. The application of discrete element simulation analysis to apple, banana, rape, and other crop fertilization machinery provides a basis for studying fertilization machinery design and fertilizing performance in mango orchards. In this study, a small spiral fertiliser apparatus was designed according to the agronomic characteristics of fertilization in mango orchards. The fertilizing performance test and parameter optimization of the spiral fertiliser discharger were carried out by adopting the bench test and simulation test. Firstly, the main influencing factors of the fertilizing performance of the fertiliser discharger were analysed by theoretical calculation, and the ranges of values were preliminarily determined. Meanwhile, the digital and discrete element models of the spiral fertiliser discharger were established. Then, by taking the variable coefficient of fertilizing stability as the evaluation index, the single factor and quadratic regression orthogonal rotation combination design test method were exploited to simulate the fertilizing performance, and the parameters significantly affecting the variable coefficient of fertilizing stability were explored. Subsequently, the second-order regression mathematical model of the variable coefficient of fertilizing stability and the significant parameters was established, and the genetic algorithm was adopted to optimize the regression model to obtain the optimal parameter combination. Finally, the accuracy of discrete element simulation test results of the spiral fertiliser discharger was verified by different fertiliser flowability and applicability tests, which provide a reference for the development of mango orchard fertilization machinery and related experimental research. So as to reduce the labor intensity of fruit farmers, improve fertilization efficiency and fertilization quality, as well as mango quality and yield.

## 2 Design and parameterisation of spiral fertiliser discharger

### 2.1 Structure design and working principle

**Figure 1:**
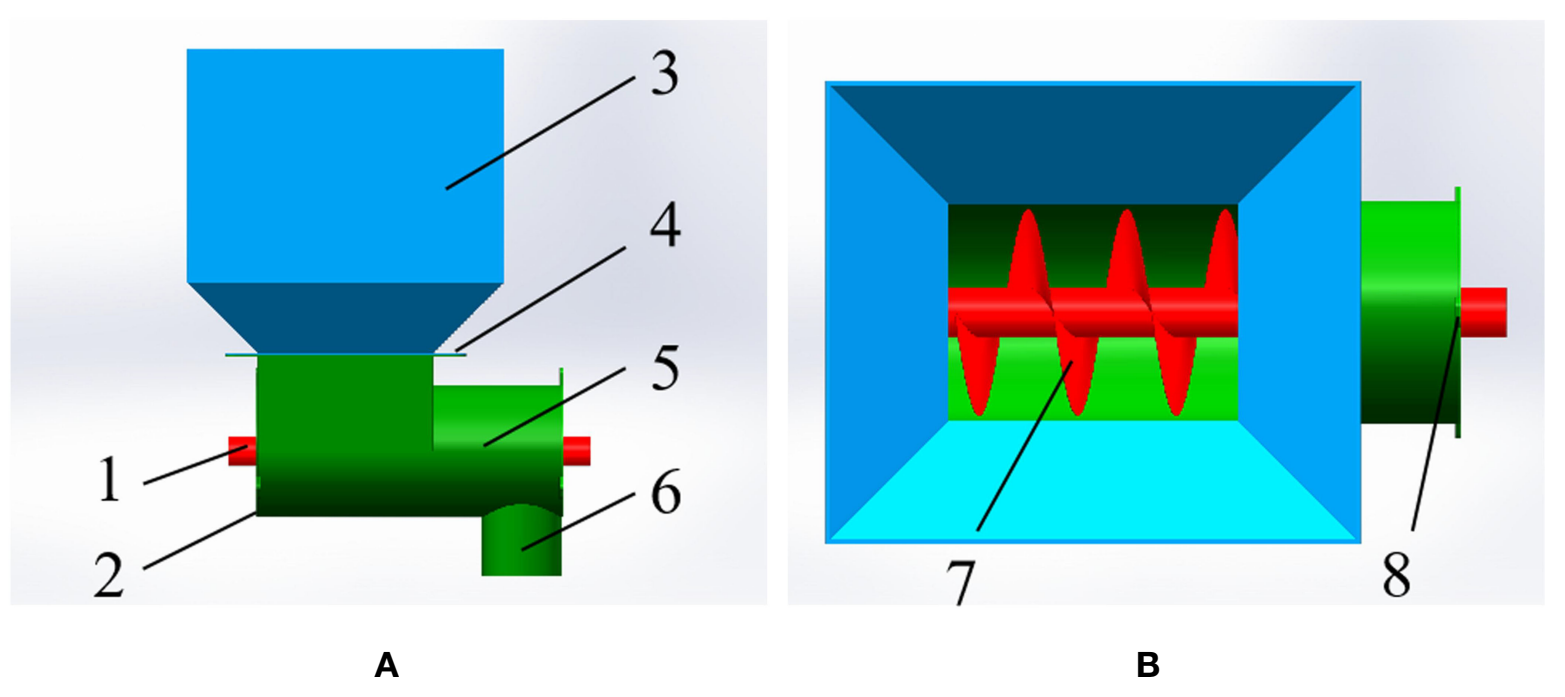
The three-dimensional diagram of spiral fertiliser discharger for the mango orchard: **(A)** the main view, **(B)** the top view, fertilizing shaft 2. left cover plate 3. fertiliser box 4. connection plate 5. fertilizing box 6. fertilizing pipe 7. spiral blade 8. right cover plate.

In this study, a small spiral fertiliser discharger was designed according to the agronomic characteristics of mango orchard fertilization. It is mainly composed of a fertiliser box, a fertilizing box, a spiral blade, a fertilizing shaft, and a fertilizing pipe. The three-dimensional model is shown in **Figure 1**. Specifically, the three-dimensional size of the fertiliser box is 280 mm × 260 mm × 270 mm, the inclined plate angle is set to 45°, the length of the fertiliser box is 260 mm, the diameter of the fertilizing box is 105 mm, the diameter of the fertilizing shaft is 25 mm, and the diameter of the fertilizing pipe is 65 mm. Additionally, the parameters such as the diameter of the spiral blade, the pitch, and the rotational speed of the fertilizing shaft will be calculated according to the agronomic requirements of the mango orchard and the relevant theoretical formulas. The main working process of the spiral fertiliser discharger is introduced as follows. Firstly, an appropriate amount of fertiliser is poured into the fertiliser box, and then the speed-regulating motor is started to drive the fertilizing shaft and the spiral blade to rotate. Under the drive of the two, the fertiliser is orderly transported to the right and falls into the fertiliser pipe and soil, thus completing the fertilization operation.

### 2.2 Determination of the main parameters of the fertiliser discharger

#### 2.2.1 The fertilizing amount

The fertilizing amount is a key index to evaluate the performance of the spiral fertiliser discharger, and it is related to the structure and motion parameters of the fertiliser discharger. In the process of fertiliser application, the cross-sectional area of the fertilizing shaft affects the performance of the fertiliser discharger. However, compared with the whole fertiliser discharger, the shaft diameter is small, and its axial blocking effect is often ignored. Therefore, the fertilizing amount discharged by the spiral fertiliser discharger can be approximately calculated as:


(1) $$Q=47D^{2}\cdot n\cdot S\cdot \lambda \cdot \epsilon \cdot \phi$$

where, *Q* is the fertilizing amount (unit: t/h), *D* is the diameter of the spiral blade (unit: mm), *n* is the rotational speed of the fertilizing shaft (unit: r/min), *S* is the pitch (unit: mm), *λ* is the fertiliser density (unit: t/m^3^), *ϵ* is the coefficient of inclined conveying, and *ϕ* is the filling factor.

From the formula (1), it can be seen that the fertilizing amount *Q* f the spiral fertiliser discharger is related to *D*, *n*, *S*, *λ*, *ϵ*, *ϕ*, etc. When the fertilizing amount *Q* is determined, parameters such as the diameter of the spiral blade *D*, the rotational speed of the fertilizing shaft *n*, and the pitch *S* can be adjusted appropriately to meet the demand for the fertilizing amount per mango tree. When the fertiliser spreader travels continuously forward one plant distance, the fertilizing amount of the spiral fertiliser discharger can be calculated as:


(2) $$Q=\frac{v\cdot f}{R}$$

where, *v* is the forward speed of the fertiliser applicator (unit: km/h); *f* is the fertilizing amount per mango tree (unit: kg/plant), and *R* is the average spacing length of mango trees (unit: m).

**Figure 2:**
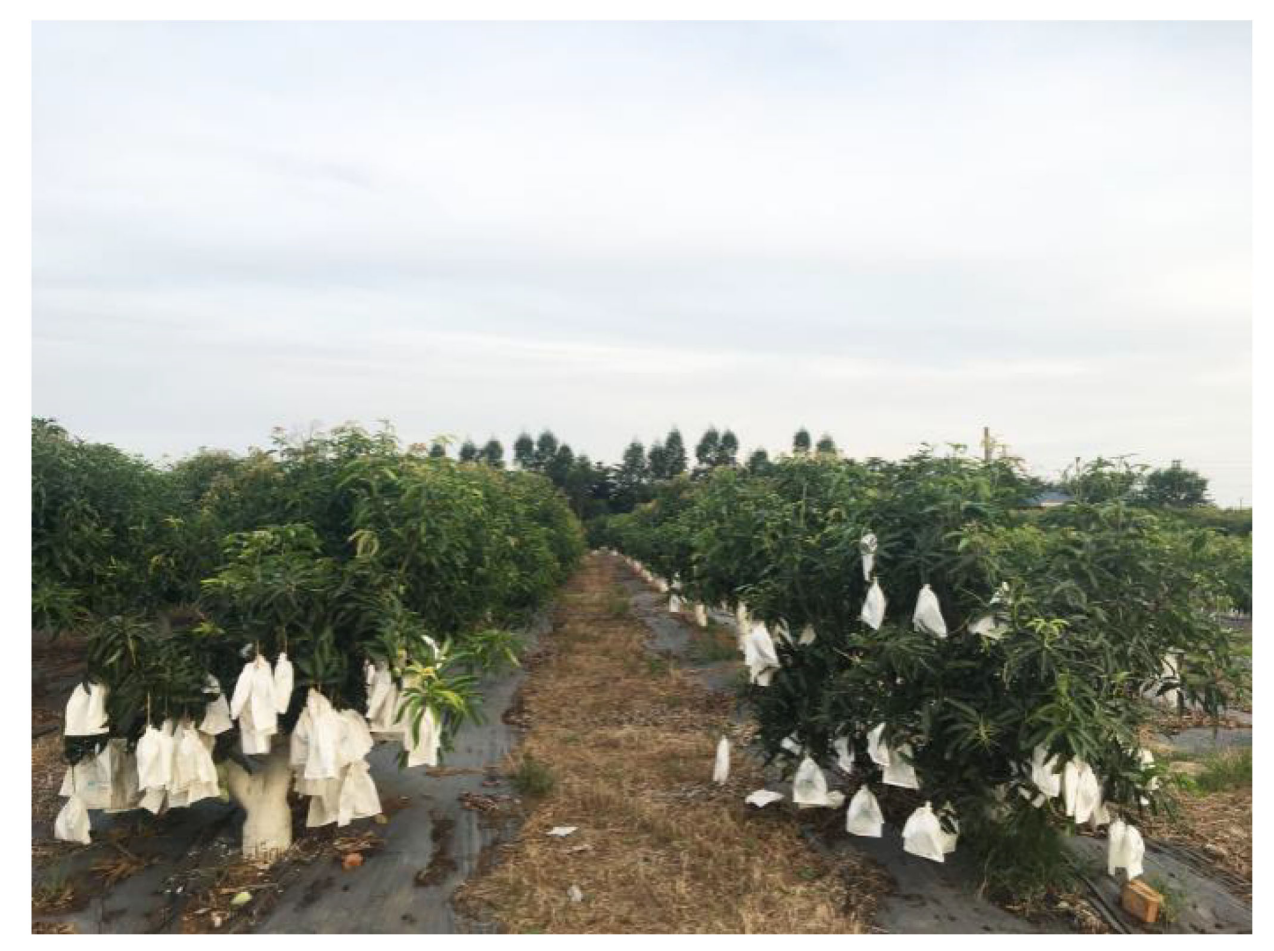
Mango orchard planting situation.


In this study, the forward speed *v* of the fertiliser discharger was set to 2 km/h. The national standard ‘Mango Cultivation Technical Regulations’ indicates that the fertilizing amount *f* during the topdressing period of mango trees is generally 0.3-0.5 kg/plant. Meanwhile, the average plant spacing and row spacing of mango trees were measured through on-the-spot investigation. As shown in **Figure 2**, the average plant spacing *R* was finally measured to be 1.82 m, and the average row spacing was 3.16 m. Then, the above data are substituted into Formula (2) to calculate the fertilizing amount required for mango orchards.

(3) $$Q=\frac{v\cdot f}{R}=0.329\sim 0.549t/h$$

#### 2.2.2 The diameter of the spiral blade and pitch

The diameter of the spiral blade is an important structural parameter of the spiral fertiliser discharger, and it directly affects the fertilizing performance. The diameter of the spiral blade is usually determined according to the structure of the fertiliser discharge device, the physical and mechanical properties of the fertiliser, and the fertilizing amount required of fruit trees. In this study, the diameter of the spiral blade of the fertiliser discharger is calculated by Formula (4).


(4) $$Q=47K_{1}\cdot J\cdot \lambda \cdot \epsilon \cdot \phi \cdot D^{\frac{5}{2}}$$

Let


(5) $$K={\left(\frac{1}{47K_{1}J}\right)}^{\frac{2}{5}}$$

Therefore,


(6) $$D=K{\left(\frac{Q}{\lambda \cdot \epsilon \cdot \phi }\right)}^{\frac{2}{5}}$$

where, *K*_1_ is the ratio coefficient between the diameter of the spiral blade and pitch, and is usually 0.5-0.9 for a horizontally arranged fertiliser discharger; *J* is the fertiliser composite characteristic coefficient, and it is set to 28 with reference to **Supplementary Table 1**; *λ* is the fertiliser density, and it is measured to be 0.913 t/m^3^; *ϵ* is the inclined conveying coefficient, and it usually takes the value of 1 for the spiral fertiliser discharger designed in this study, which is installed horizontally; *ϕ* is the filling factor, and it is set to 0.2 with reference to **Supplementary Table 1**; *K* is the fertiliser synthesis factor, and it is set to 0.0632 with reference to **Supplementary Table 1**.

Then, the above data are substituted into Formula (4) to calculate as 80.08-98.2 mm. Since the diameter *D* of the spiral blade is usually a standard integer, so the diameter of the spiral blade is initially set to 80-100 mm.

Pitch is a key structural parameter of the spiral fertiliser discharger. It determines the size of the spiral lift angle and changes the movement speed and direction of the granular fertiliser, so it is closely related to the fertilizing performance of the spiral fertiliser discharger. The pitch is usually calculated according to the following empirical formula:


(7) $$S=K_{1}\cdot D$$

#### 2.2.3 The rotational speed of the fertilizing shaft

The rotational speed of the fertilizing shaft significantly affects the fertilizing performance of the spiral fertiliser discharger. Usually, the greater the speed of the fertilizing shaft, the larger the fertilizing amount of the fertiliser discharger. However, the speed of the fertilizing shaft should not be too large because when the speed of the fertilizing shaft exceeds a critical value, excessive centrifugal force will be generated to throw the fertiliser outwards, resulting in the inability to discharge fertiliser normally, so the speed of the fertilizing shaft needs to be limited not greater than the critical value. The speed of the fertilizing shaft is usually determined according to the physical and mechanical properties of the fertiliser, the diameter of the spiral blade, and other parameters. When there is no radial movement of the granular fertiliser, the relationship between the maximum centrifugal force on the outside of the spiral and its gravity is represented below.


(8) $$m\omega_{max}^{2}r\leq mg$$

That is,


(9) $$2\pi n_{max}/60\leq \sqrt{gr}$$

Considering the influence of fertiliser discharger by different physical properties of fertilisers, we have:


(10) $$\pi n_{max}/30\leq K\sqrt{gr}$$

(11) $$n_{max}=\frac{30K}{\pi }\sqrt{\frac{g}{r}}=\frac{30K}{\pi }\sqrt{\frac{2g}{D}}$$

Let $J=30K\sqrt{2g}/\pi$ . Then, eq. (11) can be translated into the common empirical formula:


(12) $$n_{max}=J/\sqrt{D}$$

where, *m* is the mass of fertiliser (unit: kg), *ω_max_* is the critical angular velocity of the fertilizing shaft (unit: rad/s), *r* is the radius of the spiral (unit: m), *n_max_* is the critical speed of fertilizing shaft (unit: r/min), and *g* is the gravitational acceleration (unit: m/s^2^).

Through calculation, the maximum rotational speed of the fertilizing shaft is 89 r/min. To satisfy the amount of fertiliser required for the mango garden, the rotational speed of the fertilizing shaft should not be too high, and it is not allowed to exceed the critical value, i.e., the following condition should be met.


(13) $$n\leq n_{max}$$

where, *n* is the actual speed of the fertilizing shaft (unit: r/min).

To sum up, the diameter of the spiral blade, the pitch, and the rotational speed of the fertilizing shaft are the key parameters affecting the fertilizing performance of the spiral fertiliser discharger. According to the agronomic characteristics of fertilization in mango orchards and the related theoretical calculation formulas, the adjustment ranges of the relevant parameters of the small spiral fertiliser discharger in mango orchards are preliminarily determined, i.e., the diameter of the spiral blade is 80-100 mm, the pitch is 50-70 mm, and the rotational speed of fertilizing shaft is 15-55 r/min. Bassed on this, the three-dimensional solid model of all parts of the spiral fertiliser discharger is established.

## 3 Materials and methods

### 3.1 Discrete element modelling of the fertiliser discharger and the granular fertiliser

Before using the discrete element simulation software EDEM to simulate the fertilizing test of the spiral fertiliser discharger, it is necessary to understand the physical and mechanical properties of the granular fertiliser. The shape and density of the granular fertiliser directly affect its movement in the fertilizing box and the fertilizing performance. To make the discrete element model of the granular fertiliser closer to the actual fertiliser, by taking the compound fertiliser commonly used in mango orchards as the object, 100 compound fertiliser particles were randomly selected, the length (*L*), width (*W*), and thickness (*T*) of the compound fertiliser particles were measured by an electronic digital vernier calliper with an accuracy of 0.01 mm, and then the measured data were collated to calculate the equivalent diameter (*D_1_*), sphericity (*Φ*), and standard deviation. After statistical analysis, the relevant parameters of the triaxial size of the compound fertiliser particles are shown in **Supplementary Table 2**, and the relevant calculation formulas are as follows.


(14) $$D_{1}=\sqrt[{3}]{LWT}$$

(15) $$\Phi =\frac{D_{1}}{L}$$

where, *D*_1_ is the equivalent diameter (unit: mm), *L* is the length (unit: mm), *W* is the width (unit: mm), *T* is the thickness (unit: mm), and *Φ* is the sphericity.

From **Supplementary Table 2**, it can be seen that the average length of the compound fertiliser particles is 4.168 mm, the average width is 3.819 mm, the average thickness is 3.535 mm, the average equivalent diameter is 3.829 mm, the average sphericity is 92.1%, and the shape of its profile is approximately ellipsoidal. Therefore, the EDEM software was exploited to build a discrete element model of the compound fertiliser particles, and the ellipsoid was set to have a long axis of 4.2 mm and a short axis of 3.6 mm. As the EDEM software cannot directly establish the ellipsoid, five spheres were used for filling to establish the discrete element model of the compound fertiliser particles, and the radius of the five spheres was set to 1.7 mm, 1.75 mm, 1.8 mm, 1.75 mm, and 1.7 mm respectively, and the discrete element model of the compound fertiliser particles is shown in **Supplementary Figure 1**.

Then, the 3D design software SolidWorks was used to build the digital model of the spiral fertiliser discharger and imported into the solving environment of the EDEM software in the igs format. The relevant physical and mechanical parameters of the compound fertiliser particles and carbon structural steel Q235 were obtained by actual measurements with instruments such as the WD-E micro-controlled electronic universal testing machine, the MV-VS078FM high-speed photographic instrument, and the MXD-01 friction coefficient instrument, as listed in **Supplementary Table 3**. Meanwhile, the material of all components of the spiral fertiliser discharger was set to be carbon structural steel Q235, and the relevant simulation parameters of the compound fertiliser particles were set.

### 3.2 Contact model selection

**Figure 3:**
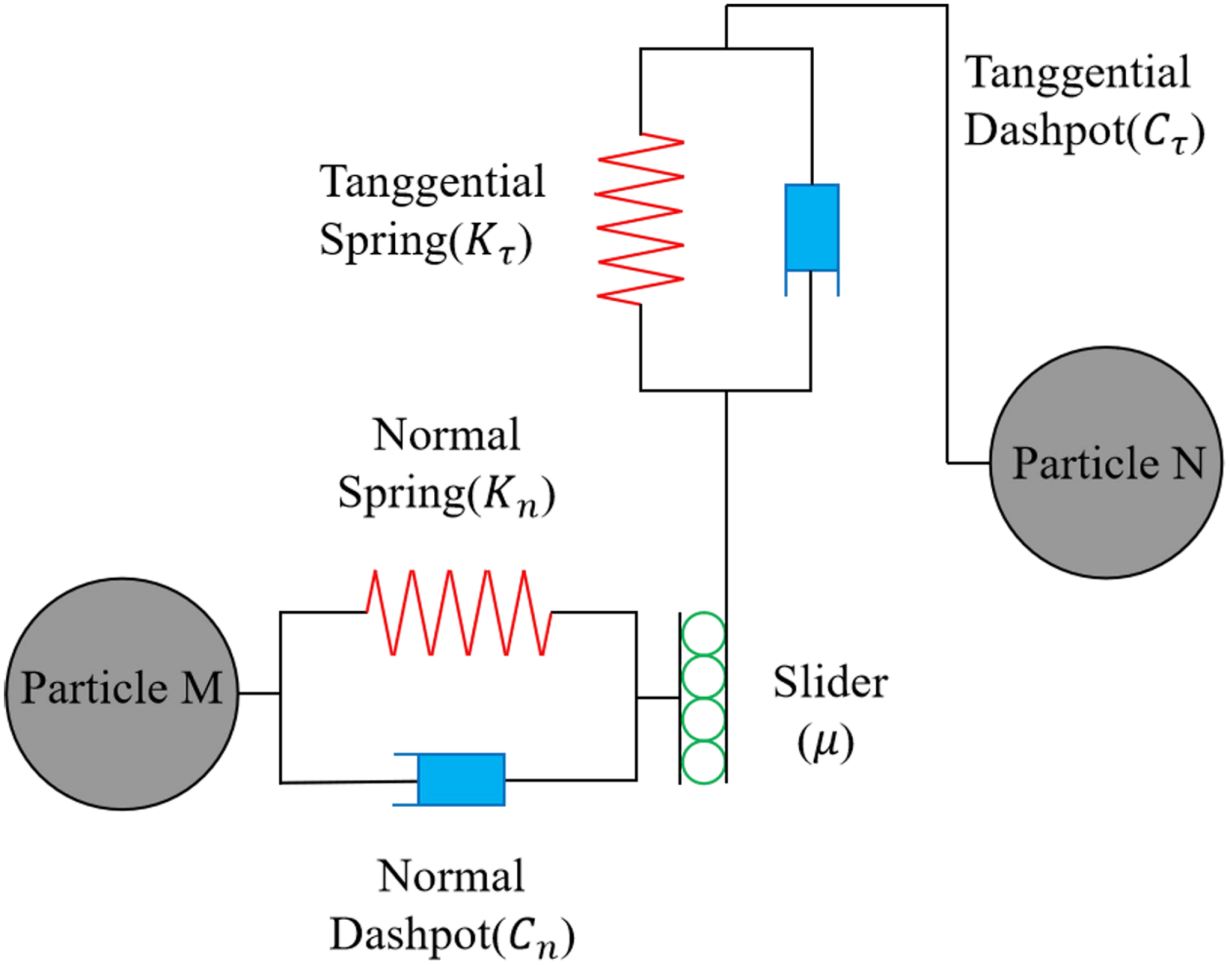
The working principle of the Hertz-Mindlin no-slip contact mechanics model.

The contact model is usually selected according to the characteristics of the study object, and the main contact models include the Hertz-Mindlin, Hertz-Mindlin with JKR, Hertz-Mindlin with bonding, etc. Different contact models have different application scenarios. According to the physical characteristics of granular fertilisers and their low surface adhesion, and assuming that the force, velocity, and displacement changes of granular fertilisers in the fertiliser discharge process are determined by the small elastic deformation between granular fertilisers or between granular fertilisers and geometry, and combined with Newton’s second law, each granular fertiliser moves under the action of torque and force. Therefore, this study selected the Hertz-Mindlin no-slip contact mechanics model for the simulation tests of fertilization. The model provides an accurate representation of the physical situation, and the mechanical calculations are accurate and efficient, which can shorten the simulation time and improve the simulation efficiency (Zhao et al., 2022). The working principle of the model is shown in **Figure 3**.

Based on the Hertz-Mindlin no-slip contact mechanics model, during discharge application, the granular fertiliser is mainly subject to gravity, granular fertiliser normal force *F_n_*, tangential force $F_{\tau }$ , normal damping force $F_{d}^{n}$ , and tangential damping force $F_{d}^{\tau }$ , which is represented as follows:


(16) $$F_{n}=\frac{4}{3}E^{*}\sqrt{R^{*}}\delta_{n}^{\frac{3}{2}}$$

(17) $$F_{\tau }=-S_{\tau }\delta_{\tau }$$

(18) $$F_{d}^{n}=-2\sqrt{\frac{5}{6}}\beta \sqrt{S_{n}m^{*}}v_{n}^{\bar{rel}}$$

(19) $$F_{d}^{\tau }=-2\sqrt{\frac{5}{6}\beta }\sqrt{S_{\tau }m^{*}}v_{\tau }^{\bar{rel}}$$

with


(20) 1E*=vi21−Ei+v1−j2Ej

(21) $$\frac{1}{R^{*}}=\frac{1}{R_{i}}+\frac{1}{R_{j}}$$

(22) $$S_{\tau }=8_{G}^{*}\sqrt{R^{*}\delta_{n}}$$

(23) $$\beta =\frac{lne}{\sqrt{ln^{2}e+\pi^{2}}}$$

(24) $$S_{n}=2_{E}^{*}\sqrt{R^{*}\delta_{n}}$$

where, $E^{*}$ is the equivalent modulus of elasticity; $R^{*}$ is the equivalent radius; $\delta_{n}$ is the normal overlap; $E_{i}$ and $E_{j}$ are the modulus of elasticity; $v_{i}$ and $v_{j}$ are Poisson’s ratio; $R_{i}$ and $R_{j}$ are the radius of contact particles; $S_{\tau }$ is the tangential stiffness; $\delta_{\tau }$ is the tangential overlap; $G^{*}$ is the equivalent shear modulus, $S_{n}$ is the normal stiffness; $m^{*}$ is the equivalent mass; *e* is the recovery factor; β is the damping ratio; $v_{n}^{\bar{rel}}$ is the normal component of relative velocity; $v_{\tau }^{\bar{rel}}$ is the tangential component of relative velocity.

The tangential force $F_{\tau }$ between granular fertilisers and between granular fertilisers and geometry is limited by Coulomb friction $\mu_{s}F_{n}$ ; meanwhile, granular fertilisers are susceptible to rolling friction during discharge application, which can be expressed in terms of moments $T_{i}$ on the contact surfaces of granular fertilisers as follows.


(25) $$T_{i}=\mu_{r}F_{n}R_{i}\omega_{i}$$

where, $\mu_{s}$ is the static friction factor, $\mu_{r}$ is the rolling friction factor, and $\omega_{i}$ is the unit angular velocity vector of the granular fertiliser at the point of contact.

The granular fertiliser moves under torque and force. When the tangential force is greater than the Coulomb friction, the granular fertiliser slides; therefore, the tangential moment and the rolling friction moment together determine the motion of the granular fertiliser.

### 3.3 Simulation of the fertiliser discharge test method

**Figure 4:**
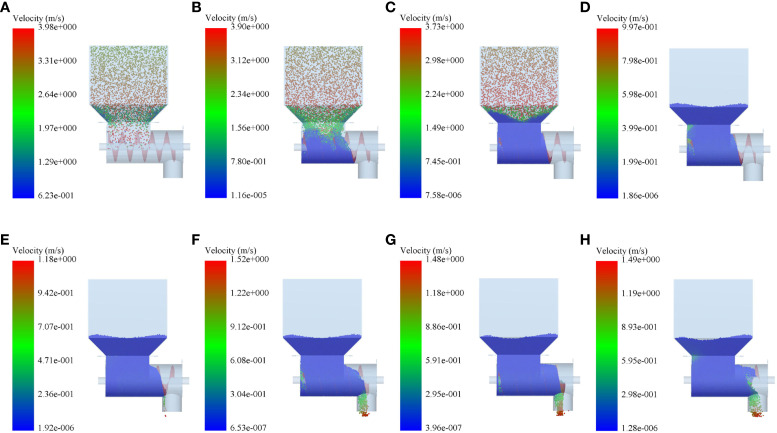
The process of simulating fertiliser discharge: **(A)** 0.1 s, **(B)** 0.6 s, **(C)** 1.1 s, **(D)** 2.2 s, **(E)** 2.8 s, **(F)** 3.7 s, **(G)** 5.3 s, **(H)** 6.0 s.

Before the simulation fertilizing test, the relevant simulation parameters of the granular fertiliser and the spiral fertiliser discharger were first set up, and a rectangular granular plant was established at the position directly above the fertiliser box for granular fertiliser generation, and granular fertiliser was generated dynamically in a fixed form to improve the efficiency of simulating fertiliser discharge, with 80,000 and 40,000 fertiliser granules being generated per second and the total simulation time of 6 s. In the fertiliser discharge process, the fertiliser granules are free to fall in the -Z-axis direction at a speed of 2 m/s. When all the fertiliser granules are generated and fall into the fertiliser box and the fertilizing box, the spiral blade and the fertilizing shaft are set to rotate at the corresponding speed. Then, under the drive of the spiral blade and the fertilizing shaft, the granular fertiliser is transported to the right in an orderly manner and falls into the fertiliser discharge tube, and a fertiliser discharge monitoring area is set up at the outlet of the fertiliser discharge tube. The mass flow rate of the granular fertiliser can be obtained through the EDEM post-processing module after the end of the simulation of fertiliser discharge, thus completing the whole simulation of fertiliser discharge test of the spiral fertiliser discharger. The process of simulating fertiliser discharge is shown in **Figure 4**.

(1) Single-factor test. According to the previous theoretical analysis of the spiral fertiliser discharger, the main factors affecting the performance of the spiral fertiliser discharger are the diameter of the spiral blade, the pitch, the rotational speed of the fertilizing shaft, etc. Further single-factor tests were conducted to verify the performance of the spiral fertiliser discharger. In this study, the diameter of and spiral blade *A*, the pitch *B*, and the rotational speed of the fertilizing shaft *C* were used as test factors, and the variable coefficient *V* of fertilizing stability was used as the response value to perform a single-factor simulation test of fertiliser discharge performance. Each group of trials was repeated three times, the variable coefficient of fertilizing stability was obtained by the following formulas, and the factor levels for the single-factor simulation of fertiliser discharge trials are shown in **Table 1**.


(26) $$\delta =\frac{\sum_{x=1}^{y}Q_{n}}{x}$$

(27) $$\sigma =\sqrt{\frac{\sum_{x=1}^{y}{\left(Q_{n}-\delta \right)}^{2}}{x}}$$

(28) $$V=\frac{\sigma }{\delta }\times 100\%$$

where, *δ* is the average fertilizing amount for all data in the fertiliser monitoring area (unit: g), *Q_n_* is the fertilizing amount from a particular trial collected in the fertiliser monitoring area (unit: g); *x* is the number of trials; *y* is the total number of data points collected in the fertiliser monitoring area; *σ* is the standard deviation of the fertilizing amount for all data in the fertiliser monitoring area, and *V* is the variable coefficient of fertilizing stability (unit: %).

**Table 1:** The factor levels for the single-factor simulation fertilizing trial.

Horizontal	The diameter of the spiral blade A (mm)	The pitch B (mm)	The rotational speed of fertilizing shaft C (r/min)
1	80	50	15
2	85	55	25
3	90	60	35
4	95	65	45
5	100	70	55

(2) Quadratic regression orthogonal rotational combination design test. To reduce the fertiliser discharge pulsation degree of the spiral fertiliser discharger and improve the fertilizing stability, based on the results of the single-factor simulation fertilizing test, the quadratic regression orthogonal rotational combination design test was carried out by using the response surface method (RSM) to further investigate the influence of the diameter of the spiral blade *A*, the pitch *B*, and the rotational speed of the fertilizing shaft *C* on the fertilizing performance of the spiral fertiliser discharger, with the variable coefficient *V* of fertilizing stability as the evaluation index. The test parameters were taken at five levels, namely, high, upper, middle, lower, and low, expressed in the form of codes +1.682, 1, 0, -1, and -1.682, respectively, and the factor level codes of the multi-factor simulation fertilizing test are shown in **Table 2**. Further, a second-order regression mathematical model between the variable coefficient of fertilizing stability and the significance parameter was established based on a quadratic regression orthogonal rotational combination design test, which provides a theoretical basis for optimising the parameters of the spiral fertiliser discharger and improving its fertilizing performance.

**Table 2:** The factor level codes for multi-factor simulation fertilizing trials.

Normative variables	Natural variables		
	The diameter of the spiral blade A (mm)	The pitch B (mm)	The rotational speed of the fertilizing shaft C (r/min)
Upper asterisk arm γ (+1.682)	100	70	55
Upper level 1	96	66	47
Zero level 0	90	60	35
Lower level -1	84	54	23
Lower asterisk arm −γ (-1.682)	80	50	15

## 4 Results and discussion

### 4.1 Single-factor test

#### 4.1.1 The diameter of the spiral blade

**Figure 5:**
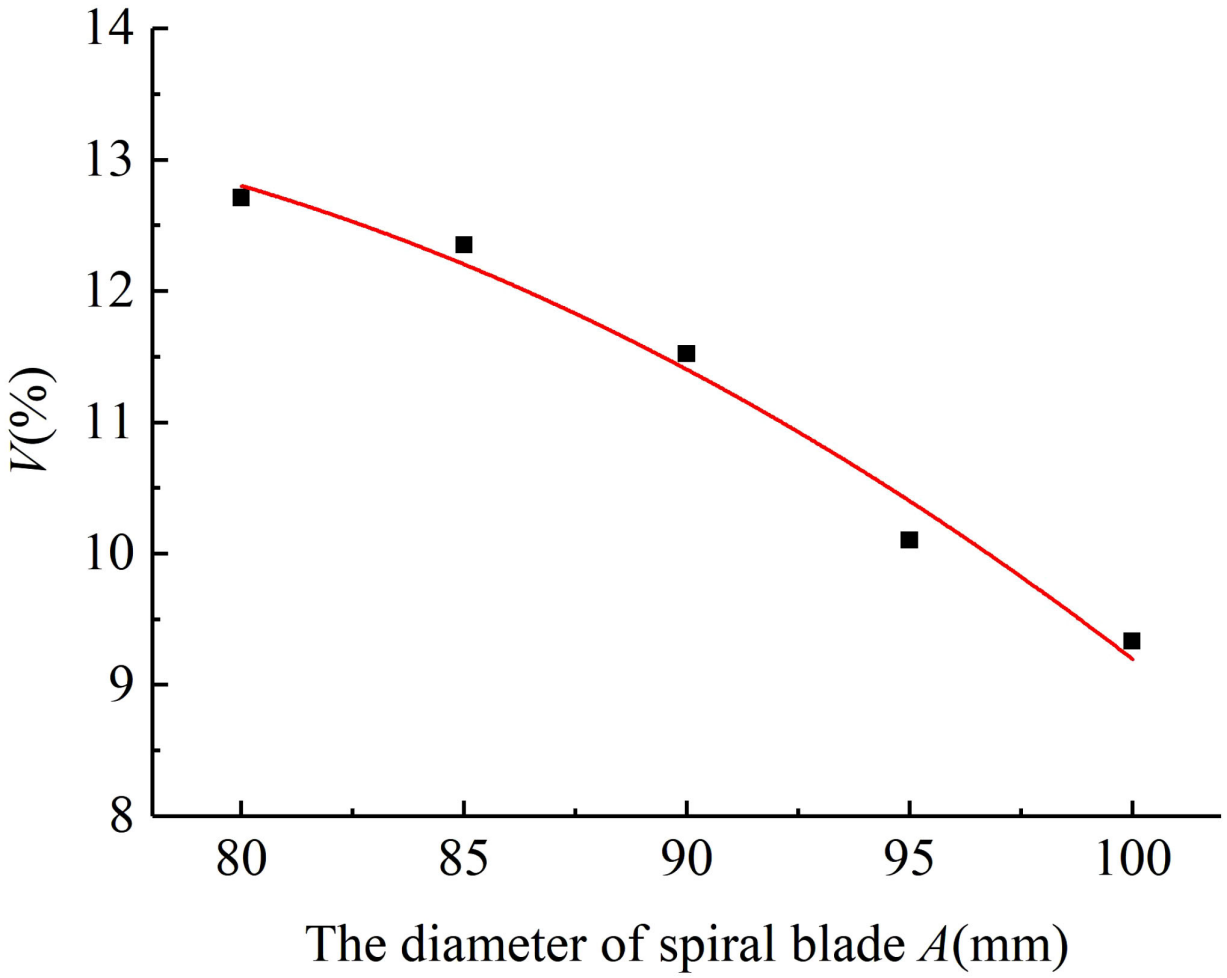
The regression fitting curve between the diameter of the spiral blade and the variable coefficient of fertilizing stability.

The variable coefficient of fertilizing stability decreased with the increase in the diameter of the spiral blade when the pitch was 60 mm and the rotational speed of the fertilizing shaft was 35 r/min. The results of the simulated fertiliser discharge test are shown in **Figure 5**. A polynomial function was used to fit the diameter of the spiral blade and the variable coefficient of fertilizing stability, and a regression curve between the two was obtained. The analysis of the variance and significance test results on the regression mathematical model and coefficients showed that the diameter of the spiral blade had a significant effect on the variable coefficient of fertilizing stability (P< 0.05), the fit of the regression model R^2^ = 0.963 indicated a good curve fit, and the diameter of the spiral blade was negatively correlated with the variable coefficient of fertilizing stability. The fitting equation is shown in Equation (29), and the standard errors of -5.01, 0.545, and 0.004 are 2.37, 0.53, and 0.003 respectively. It can be seen that the standard errors of each parameter in the model are small, indicating that the established mathematical model is accurate and reliable.


(29) $$V=-5.01+0.545A-0.004A^{2}$$

#### 4.1.2 The pitch

**Figure 6:**
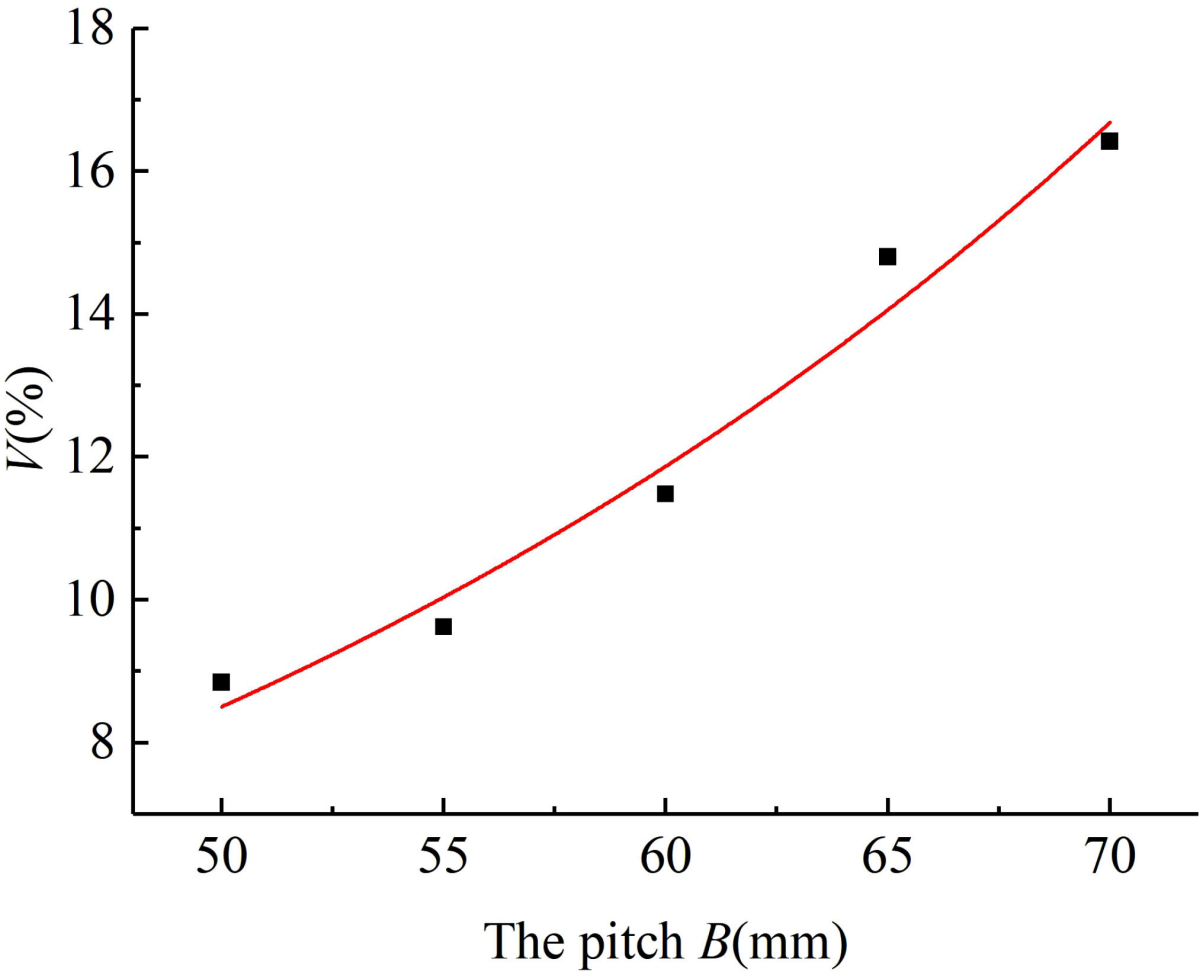
The regression fitting curve between the pitch and the variable coefficient of fertilizing stability.

The variable coefficient of fertilizing stability increased with the pitch when the diameter of the spiral blade was 90 mm and the rotational speed of the fertilizing shaft was 35 r/min. The results of the fertiliser discharge simulation are shown in **Figure 6**. An exponential function was used to fit the pitch and the variable coefficient of fertilizing stability, and the regression curve between them was obtained. The analysis of variance and significance test results on the regression model and coefficients indicated that the effect of pitch on the variable coefficient of fertilizing stability was very significant (P< 0.01), and the fit of the regression mathematical model R^2^ = 0.956 demonstrated a good curve fit and a positive correlation between the pitch and the variable coefficient of fertilizing stability, The fitting equation is shown in Equation (30), and the standard errors of 0.572, 0.029, and 0.00003 are 2.45, 0.08, and 0.0006 respectively. It can be seen that the standard errors of each parameter in the model are small, indicating that the mathematical model is accurate and reliable.


(30) $$V=\exp0.572+0.029B+0.00003B^{2}$$

#### 4.1.3 The rotational speed of the fertilizing shaft

**Figure 7:**
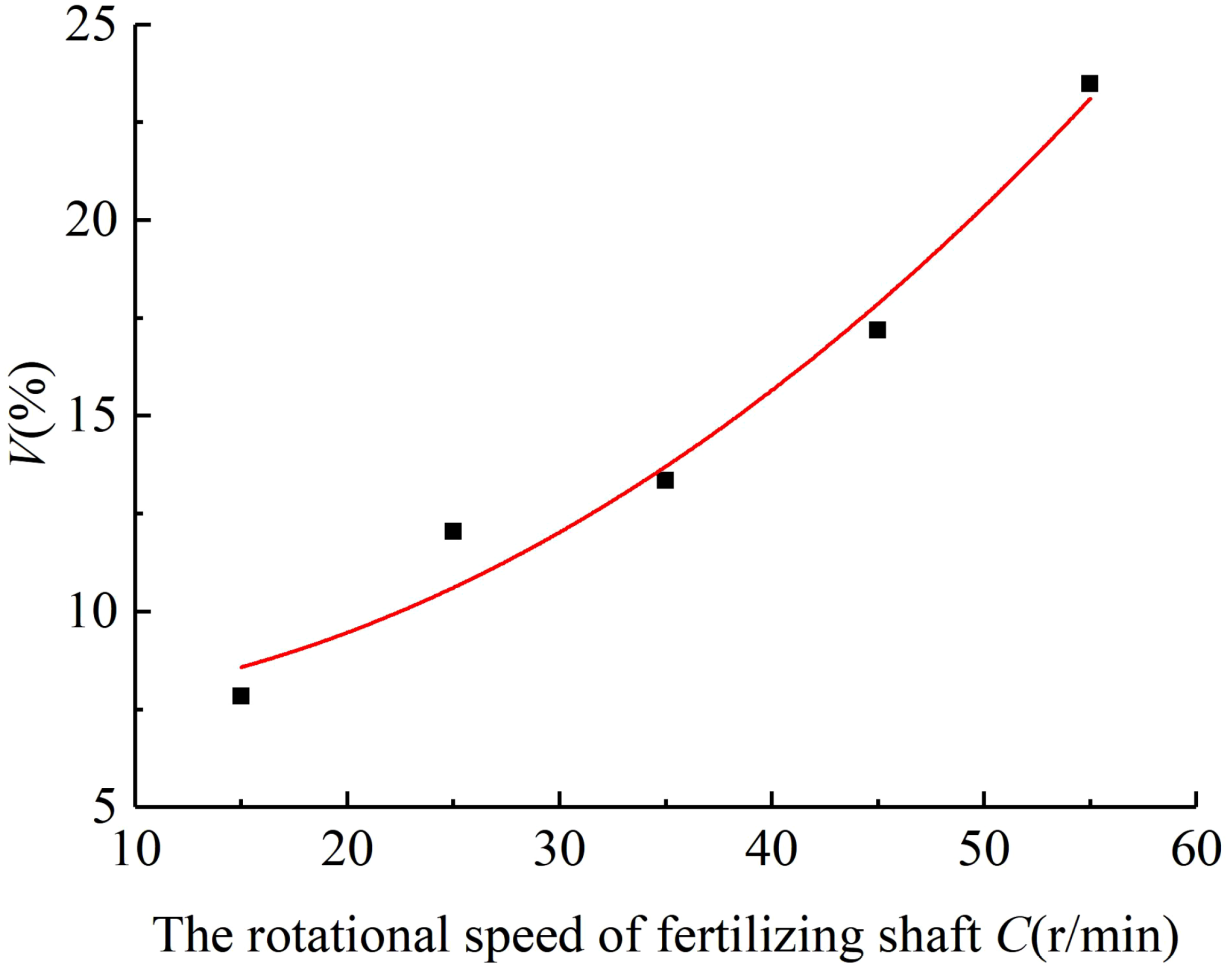
The regression fitting curve between the fertiliser discharge shaft speed and the variable coefficient of fertilizing stability.

When the diameter of the spiral blade was 90 mm and the pitch was 60 mm, the variable coefficient of fertilizing stability increased with the rotational speed of the fertilizing shaft, and the results of the fertiliser discharge simulation are shown in **Figure 7**. The regression curve was obtained by fitting a power model to the rotational speed of the fertilizing shaft and the variable coefficient of fertilizing stability. Then, the regression mathematical model and coefficients were analysed by ANOVA (analysis of variance) and significance test. It can be seen that the influence of the rotational speed of the fertilizing shaft on the variable coefficient of fertilizing stability was very significant (P< 0.01), the fit of the regression model R^2^ = 0.952 indicated a good fitting, and the rotational speed of the fertilizing shaft was positively correlated with the variable coefficient of fertilizing stability. The fitting equation is shown in Equation (31), and the standard errors of 7.463, 0.005, and 2.032 are 2.15, 0.01, and 0.73 respectively. It can be seen that the standard errors of each parameter in the model are small, indicating that the mathematical model has high accuracy and reliability.


(31) $$V=7.463+0.005C^{2.032}$$

To sum up, the results of the single-factor simulated fertilizing performance tests show that the diameter of the spiral blade, the pitch, and the rotational speed of the fertilizing shaft significantly affect the variable coefficient of fertilizing stability of the spiral fertiliser discharger. Especially, the diameter of the spiral blade is negatively correlated with the variable coefficient of fertilizing stability, and the pitch and the rotational speed of fertilizing shaft are positively correlated with the variable coefficient of fertilizing stability. These results verify the reliability of the theoretical analysis of the spiral fertiliser discharger in the previous study.

### 4.2 Quadratic regression orthogonal rotational combination design trial

According to factor level codes in **Table 2**, the multi-factor simulated fertilizing test scheme was determined by using the Design-Expert software. Specifically, Nine central points were used for error estimation in the simulated fertilizing test, a total of 23 sets of tests were conducted, and each set of tests was repeated three times. The corresponding variable coefficient of fertilizing stability was obtained through the calculation of formulas (26) - (28), and then a series of data analyses was conducted. The results of the multifactorial simulation fertilizing test are shown in **Table 3**.

**Table 3:** The scheme and results of the multi-factor simulation fertilizing trial.

Serial number	The diameter of the spiral blade A (mm)	The pitch B (mm)	The rotational speed of the fertilizing shaft C (r/min)	The variable coefficient of fertilizing stability V (%)
1	-1	-1	-1	8.76
2	1	-1	-1	7.31
3	-1	1	-1	9.59
4	1	1	-1	8.62
5	-1	-1	1	14.13
6	1	-1	1	12.65
7	-1	1	1	18.15
8	1	1	1	16.32
9	-1.682	0	0	12.26
10	1.682	0	0	10.09
11	0	-1.682	0	9.21
12	0	1.682	0	15.86
13	0	0	-1.682	7.12
14	0	0	1.682	21.87
15	0	0	0	11.09
16	0	0	0	11.24
17	0	0	0	12.27
18	0	0	0	11.41
19	0	0	0	12.67
20	0	0	0	13.15
21	0	0	0	11.28
22	0	0	0	12.63
23	0	0	0	11.45

#### 4.2.1 Regression model, analysis of variance and significance test

Through multiple regression analysis of the above experimental results by using the Design-Expert software, a mathematical model can be obtained for the regression between the variable coefficient of fertilizing stability and the diameter of spiral blade, the pitch, and the rotational speed of the fertilizing shaft.


(32) $$V=-74.77+2.13A-0.28B-0.49C+0.0005AB-0.0016AC+0.009BC-0.01A^{2}+0.0013B^{2}+0.005C^{2}$$

Then, the ANOVA and significance test was conducted on the regression model, and the ANOVA results of the multi-factor simulation fertilizing test in **Table 4** show that the diameter of the spiral blade *A*, the pitch *B*, the rotational speed of the fertilizing shaft *C*, and the quadratic term *C* ^2^ of the rotational speed of the fertilizing shaft have a significant effect on the variable coefficient of fertilizing stability *V*. The factors influencing the variable coefficient of fertilizing stability *V* in the order of priority are the rotational speed of the fertilizing shaft *C*, the pitch *B*, and the diameter of the spiral blade *A*. The p-value of this mathematical model is< 0.0001, indicating that the regression model of the variable coefficient of fertilizing stability is highly significant, and the misfit is not significant because the p-value of the misfit term is equal to 0.1125>0.05, indicating that the regression equation is well fitted, with the coefficient of determination R^2^ = 0.9529 and the corrected coefficient of determination R^2^_adj_ = 0.9204. Both values are close to 1, and the standard error is 0.33, indicating that the regression equation is highly reliable; meanwhile, the accuracy is 20.735, indicating that the regression mathematical model is highly accurate.

**Table 4:** The analysis of the variance results of the multi-factor simulation fertilizing test.

Source of variance	Sum of squares	Degrees of freedom	F-value	p-value	Significance
Model	250.81	9	29.25	< 0.0001	**
A	6.44	1	6.76	0.022	*
B	32.33	1	33.94	< 0.0001	**
C	196.3	1	206.07	< 0.0001	**
AB	2.11E-03	1	2.22E-03	0.9632	
AC	0.099	1	0.1	0.7523	
BC	3.85	1	4.04	0.0656	
$A^{2}$	3	1	3.15	0.0991	
$B^{2}$	0.034	1	0.035	0.8539	
$C^{2}$	8.68	1	9.11	0.0099	**
Residuals	12.38	13			
Misfit	7.64	5	2.58	0.1125	
Error	4.74	8			
Total	263.19	22			

** indicates that the term is highly significant (p<0.01), * indicates that the term is significant (p<0.05). The same notation are used below.

In the case that the regression model is significant and the misfit term is not significant, the above regression mathematical model is optimised to obtain a new equation by excluding the very insignificant term.


(33) $$V=-77.02+2.09A-0.08B-0.64C+0.0098BC-0.01A^{2}+0.0039C^{2}$$

The ANOVA results of the optimised regression mathematical model are shown in **Table 5**, which shows that the performance of the optimised regression model is improved. The coefficient of determination R^2^ = 0.9524 and the corrected coefficient of determination R^2^_adj_ = 0.9346 are close to 1, and the standard error decreased to 0.26, indicating that the reliability of the regression equation is enhanced; meanwhile, the accuracy is improved to 27.634, indicating the accuracy of the regression model is higher than that before the optimisation.

**Table 5:** The ANOVA results of the optimised model for the multi-factor simulation fertilizing test.

Source of variance	Sum of squares	Degrees of freedom	F-value	p-value	Significance
Model	250.67	6	53.4	< 0.0001	**
A	6.44	1	8.23	0.0111	*
B	32.33	1	41.33	< 0.0001	**
C	196.3	1	250.89	< 0.0001	**
BC	3.85	1	4.92	0.0413	*
$A^{2}$	3.01	1	3.85	0.0675	
$C^{2}$	8.67	1	11.08	0.0043	**
Residuals	12.52	16			
Misfit	7.78	8	1.64	0.2501	
Error	4.74	8			
Total	263.19	22			

#### 4.2.2 Response surface analysis of interaction effects

**Figure 8:**
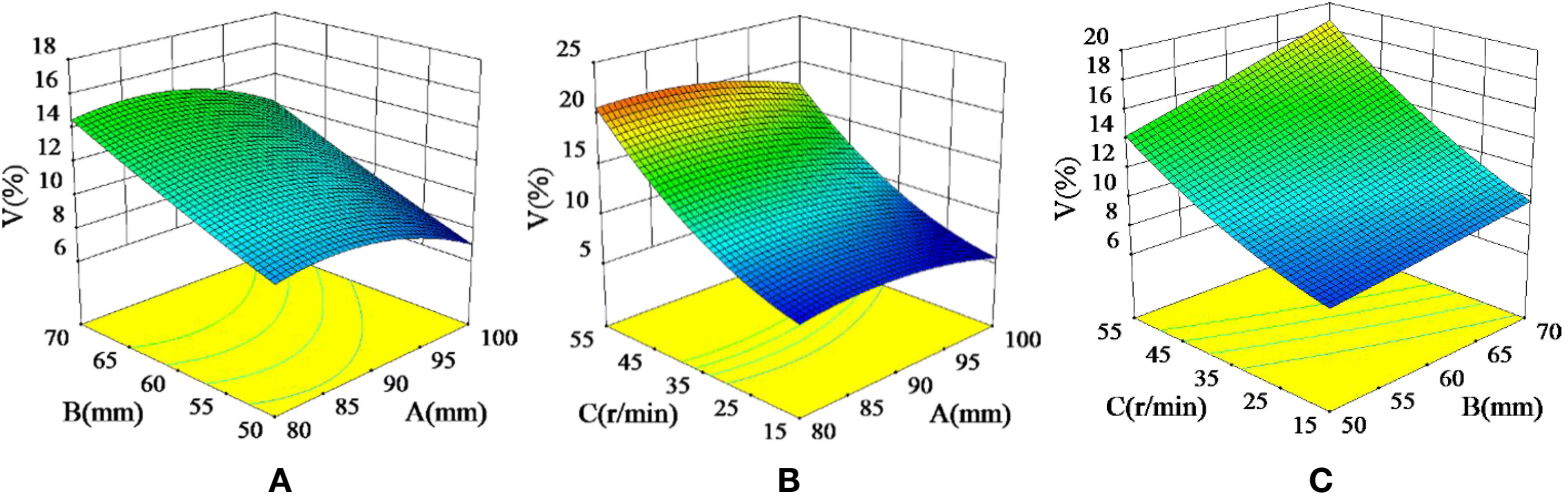
Interaction effects on the variable coefficient of fertilizing stability (*V*): **(A)** The response surface plot of the interaction between the diameter of the spiral blade and the pitch (*AB*) on the variable coefficient of fertilizing stability, **(B)** The response surface plot of the interaction between the diameter of the spiral blade and the rotational speed of the fertilizing shaft (*AC*) on the variable coefficient of fertilizing stability, **(C)** The response surface plot of the interaction between the pitch and the rotational speed of the fertilizing shaft (*BC*) on the variable coefficient of fertilizing stability.

The interactions between the test factors were analysed to investigate their effects on the fertilizing performance of the spiral fertiliser discharger. Also, the optimal parameter region was further narrowed down. **Figure 8** shows the response surface plot of the interaction effect on the variable coefficient of fertilizing stability.

**Figure 8A** shows the response surface of the interaction effect between the diameter of the spiral blade and the pitch on the variable coefficient of fertilizing stability when the rotational speed of the fertilizing shaft was 35 r/min. When the diameter of the spiral blade was 80-100 mm and the pitch was 50-70 mm, the variable coefficient of fertilizing stability decreased with the increase in the diameter of the spiral blade and increased with the increase in the pitch. The variable coefficient of fertilizing stability can be reduced by increasing the diameter of the spiral blade and decreasing the pitch. **Figure 8B** shows the response surface of the variable coefficient of fertilizing stability due to the interaction between the diameter of the spiral blade and the rotational speed of the fertilizing shaft when the pitch was 60 mm. It can be seen that the variable coefficient of fertilizing stability decreased with the increase in the diameter of the spiral blade and increased with the increase in the rotational speed of the fertilizing shaft when the diameter of the spiral blade was 80-100mm and the rotational speed of fertilizing shaft was 15-55 r/min. Therefore, the variable coefficient of fertilizing stability can be reduced by appropriately increasing the diameter of the spiral blade and decreasing the rotational speed of the fertilizing shaft. **Figure 8C** shows the response surface of the interaction between the pitch and the rotational speed of the fertilizing shaft on the variable coefficient of fertilizing stability when the diameter of the spiral blade was 90 mm, the pitch was 50-70 mm, and the rotational speed of the fertilizing shaft was 15-55 r/min. It can be seen that the variable coefficient of fertilizing stability increased with the increase in the pitch and the rotational speed of the fertilizing shaft. Thus, reducing the pitch and the rotational speed of the fertilizing shaft can reduce the variable coefficient of fertilizing stability. The results of the response surface analysis indicate that the variable coefficient of fertilizing stability increases with the pitch and the rotational speed of the fertilizing shaft.

Therefore, when the diameter of the spiral blade is 90-100 mm, the pitch is 50-60 mm, and the rotational speed of the fertilizing shaft is 15-35 r/min, the variable coefficient of fertilizing stability can be reduced by increasing the diameter of the spiral blade and decreasing the pitch and the rotational speed of the fertilizing shaft.

### 4.3 Parameters optimisation of the spiral fertiliser discharger

**Figure 9:**
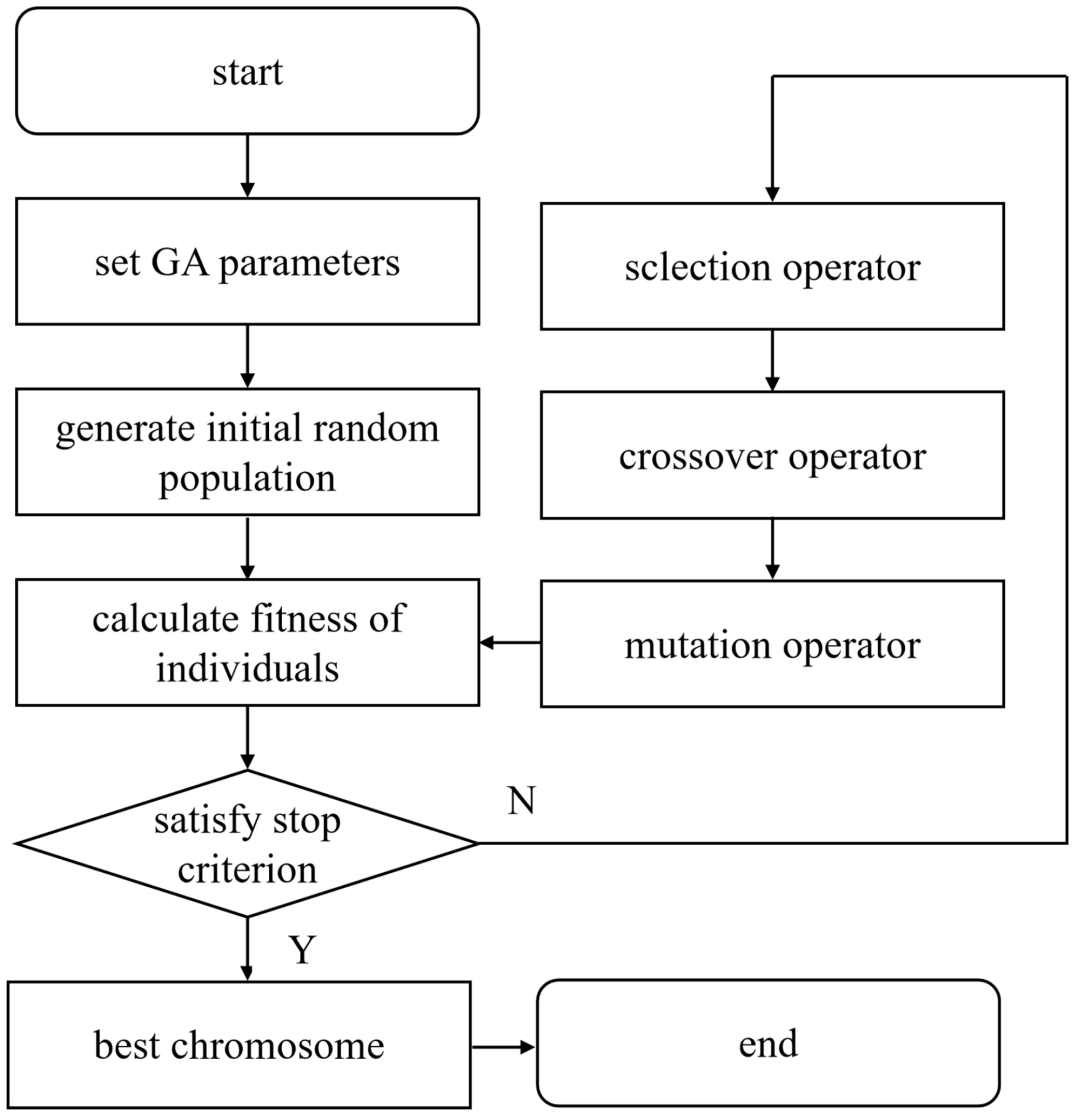
The flow chart of applying the genetic algorithm,.

The genetic algorithm (GA) is a global search algorithm based on the principle of biological genetic evolution. It simulates the phenomenon of reproduction, mating, and mutation in natural selection and genetic processes. This algorithm can overcome the shortcomings of traditional nonlinear programming algorithms that are easy to fall into local optima. It has the advantages of strong global optimization ability, robustness, and efficiency, and it has been widely used and developed (Yang et al., 2019; Bahiraei et al., 2020; Chen et al., 2021). Therefore, this study adopted the genetic algorithm to find the optimal parameter combination of the spiral fertiliser discharger. The specific process of applying the genetic algorithm is shown in **Figure 9**.

To improve the fertilizing stability of the spiral fertiliser discharger, under the premise of ensuring the safe and reliable operation of the fertiliser discharger, the variable coefficient of fertilizing stability should be as small as possible. Therefore, the objective optimization function of the variable coefficient of fertilizing stability is established, as shown in formula (34). According to the variables in the objective optimization function, the parameters to be optimized include the diameter of the spiral blade A, the pitch B, and the rotational speed of the fertiliser shaft C. Therefore, the parameter optimization problem of the spiral fertiliser discharger can be defined as finding an optimal vector X, as shown in formula (35). Meanwhile, according to the analysis results of the response surface, the variation ranges of the parameters to be optimized of the spiral fertiliser discharger are determined, and the corresponding constraint conditions are established, as shown in formula (36).


(34) V(x)=minV(A,B,C)

(35) $$X={\left[\begin{array}{ccc} A & B & C \end{array}\right]}^{T}$$

(36) $$s.t\{\begin{array}{c} 90\leq A\leq 100\\ 50\leq B\leq 60\\ 15\leq C\leq 35 \end{array}$$

In this study, the parameter optimization of the spiral fertiliser discharger is a nonlinear constrained optimization problem, which can be effectively solved by the genetic algorithm. The specific operation steps are as follows:

(1) Parameter coding. The genetic algorithm uses binary coding. There are three independent variables, namely, the spiral blade diameter A, the pitch B, and the fertilizer shaft speed C. Each independent variable is set to 6 genes, a total of 18 genes.
(2) The initial population is generated, and the population number is set to 50.
(3) Fitness evaluation function. After weighting the objective optimization function, the fitness evaluation function is defined as F(x)=1/V(x) .
(4) Genetic operation, including selection, crossover, and mutation. Copying is the basic operator of the genetic algorithm. It reproduces excellent chromosomes in the next generation of new groups, consistent with the natural selection principle of ‘ survival of the fittest’. Whether the chromosomes are copied is determined according to the size of their fitness. The larger the fitness is, the more the chromosomes are copied. Meanwhile, the chromosomes with a smaller fitness is eliminated so that the total number of chromosomes in the new group is the same as that of the original group. In this paper, the selection probability is set to 0.9, and the population is set to 50, so a total of 50 * 0.9 = 40 chromosomes are saved at each cycle. In addition, 50 * (1-0.9) = 10 optimal individuals are copied to ensure the stability of the population in the iterative process. The number of chromosomes is still 40 + 10 = 50 so that the next round of iteration is conducted. The chromosomes are selected by the roulette method. In the genetic algorithm, exchange is the main way to generate new chromosomes. It imitates the principle of hybridization in biology and exchanges some genes of the two chromosomes. The chromosomes that perform the exchange are randomly selected. First, the exchange probability is determined to be 0.75. Then, the above-mentioned roulette selection method is adopted to select the exchanged chromosomes according to the fitness size, and pairwise exchanges are performed in turn. Mutation is another method for generating new chromosomes in genetic algorithms. It mutates a character of a chromosome, such as changing the original gene 0 into 1 or changing the original gene 1 into 0. The selection of mutant chromosomes and the determination of mutation location are all generated by random methods. First, the number of chromosomes that need to be mutated is determined, e.g., the mutation probability is 0.02, and the number of populations is 50. Therefore, 50 * 0.02 = 1 chromosome is randomly mutated in each iteration, and then a position is randomly selected in the gene fragment of the chromosome to change the original gene value. The group P (t) is promoted to the next generation group P (t + 1) by three operations with guessing properties.
(5) Termination condition judgment. The maximum evolutionary algebra method is taken as the stop rule of the program. In this study, the iterative algebra 100 is set as the termination condition, i.e., the genetic operation process is terminated when 100 consecutive generations do not reproduce a new generation, and the maximum fitness individual obtained in the previous calculation process is taken as the optimal solution. The corresponding iterative convergence curve is presented in **Supplementary Figure 2**.

Through multiple iterative calculations, the optimal parameter combination of the spiral fertiliser discharger was finally obtained, i.e., 98.44 mm for the diameter of the spiral blade, 54.8 mm for the pitch, and 24.43 r/min for the rotational speed of the fertilizing shaft. In this case, the variable coefficient of fertilizing stability was 6.51%, which could meet the fertilization agronomic requirements in the national standard “Technical Specification for Quality Evaluation of Fertilization Machinery” (the total variable coefficient of fertilizing stability).

### 4.4 Experimental validation

#### 4.4.1 The flowability verification test of the granular fertiliser

**Figure 10:**
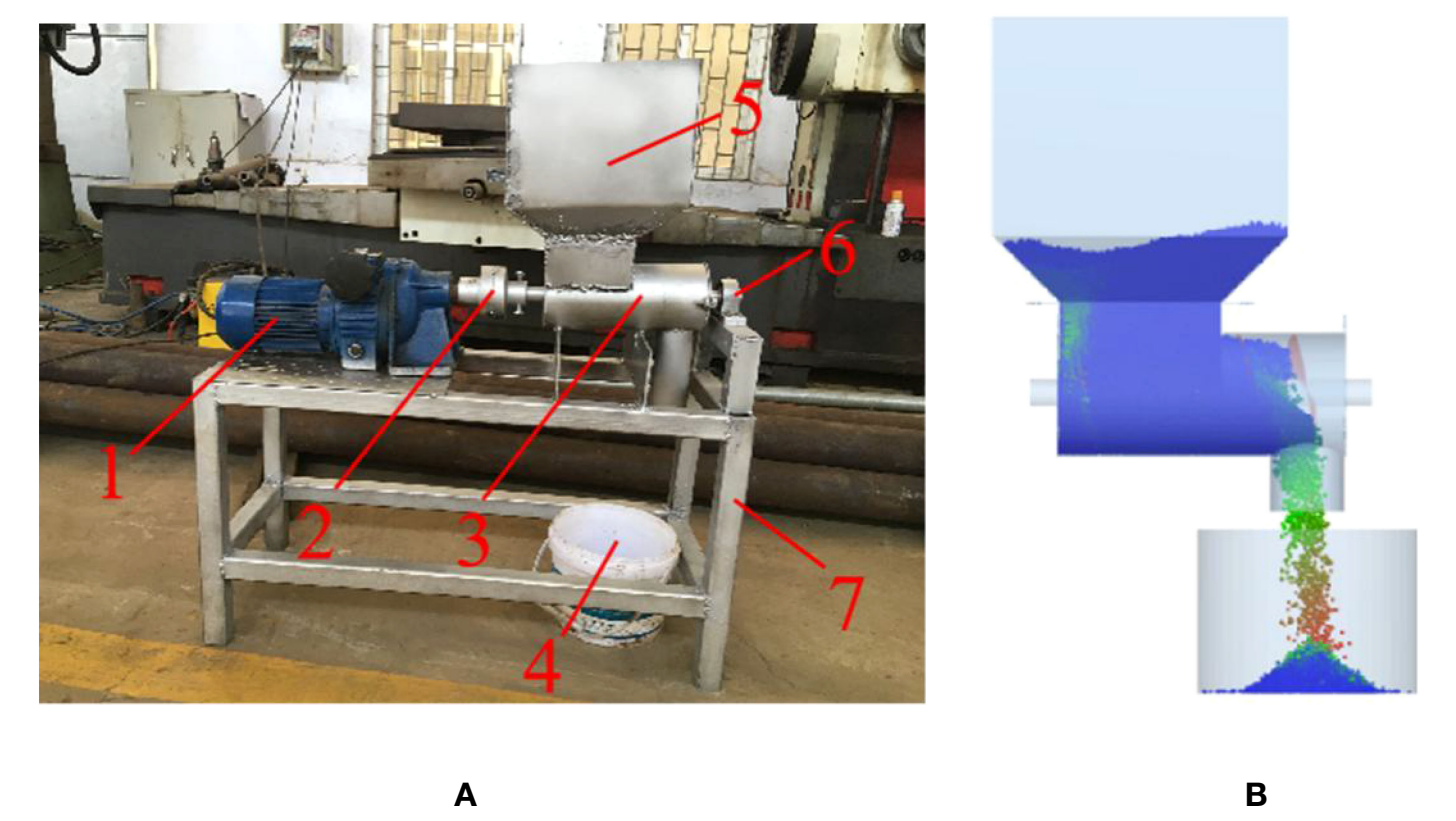
The process of granular fertiliser flow verification test: **(A)** the bench test, **(B)** the simulation test. 1. JWB-X0.37-8D type electrodeless transmission 2. coupling 3. fertilising box 4. gathering barrel 5. fertiliser box 6. bearing seat, 7. frame.

To further validate the reliability of the results of the discrete element simulation fertilizing test and the optimal parameters of the spiral fertiliser discharger, the flowability verification test of the granular fertiliser was conducted by using a combination of bench and simulation tests, the mass flow rate was taken as the evaluation index, and the measured and simulated values of the mass flow rate were compared as shown in **Figure 10**. In the fertilizing test, an appropriate amount of compound fertiliser granules are first added to the fertiliser box, and the optimal parameters of the fertiliser discharger are set; then, the power is activated, the speed-controlled motor drives the fertilizing screw to discharge the fertiliser, and the granular fertiliser drops into the collection box below the fertilizing pip. The entire process of the granular fertiliser was recorded by a camera, the flow time and the mass of the granular fertiliser in the collection box were recorded, and the mass flow rate of the granular fertiliser was calculated. The test process was repeated five times. Subsequently, the digital model of the spiral fertiliser discharger and the discrete element model of the granular fertiliser were imported into the EDEM software, and the simulated fertilizing test was carried out under the same conditions as the bench test. After this test, the mass flow rate of the granular fertiliser was derived, the average relative error between the physical value and the simulated value of the mass flow rate was calculated, and the test results were compared and analysed.

**Figure 11:**
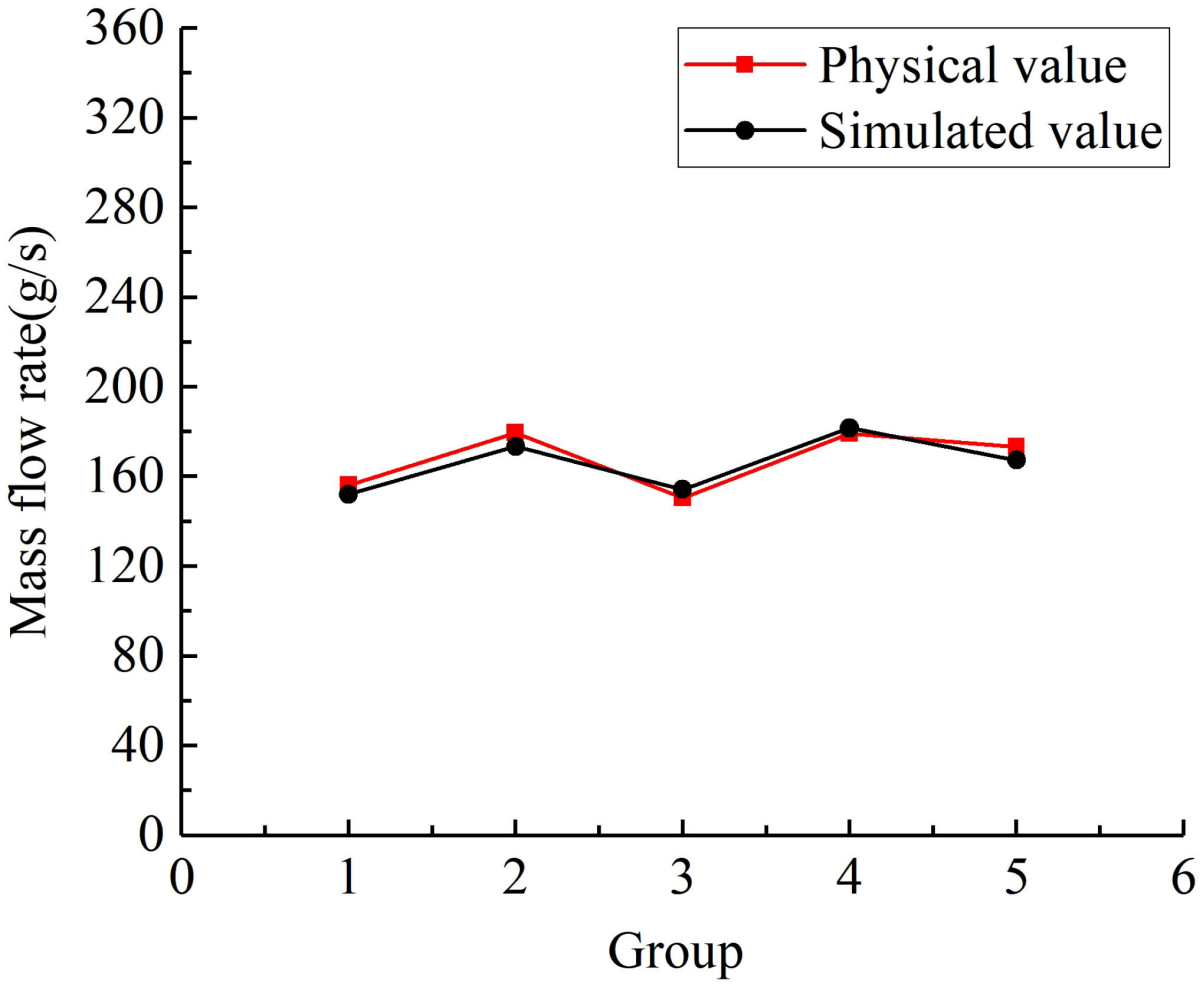
The comparison results of flowability validation test on the granular fertiliser.

**Figure 11** shows the comparison results of the flowability verification test of the granular fertiliser. The average relative error of the fertiliser flow rate between the bench and the simulated fertilization was calculated to be 2.64%. The small error indicates that there is no significant difference between the physical and simulated values of the granular fertiliser flow rate. Meanwhile, the results demonstrate the high accuracy of the regression mathematical model developed in this study and the optimal parameter combination determined for the fertiliser discharger.

#### 4.4.2 The applicability verification test of different fertilisers

**Figure 12:**
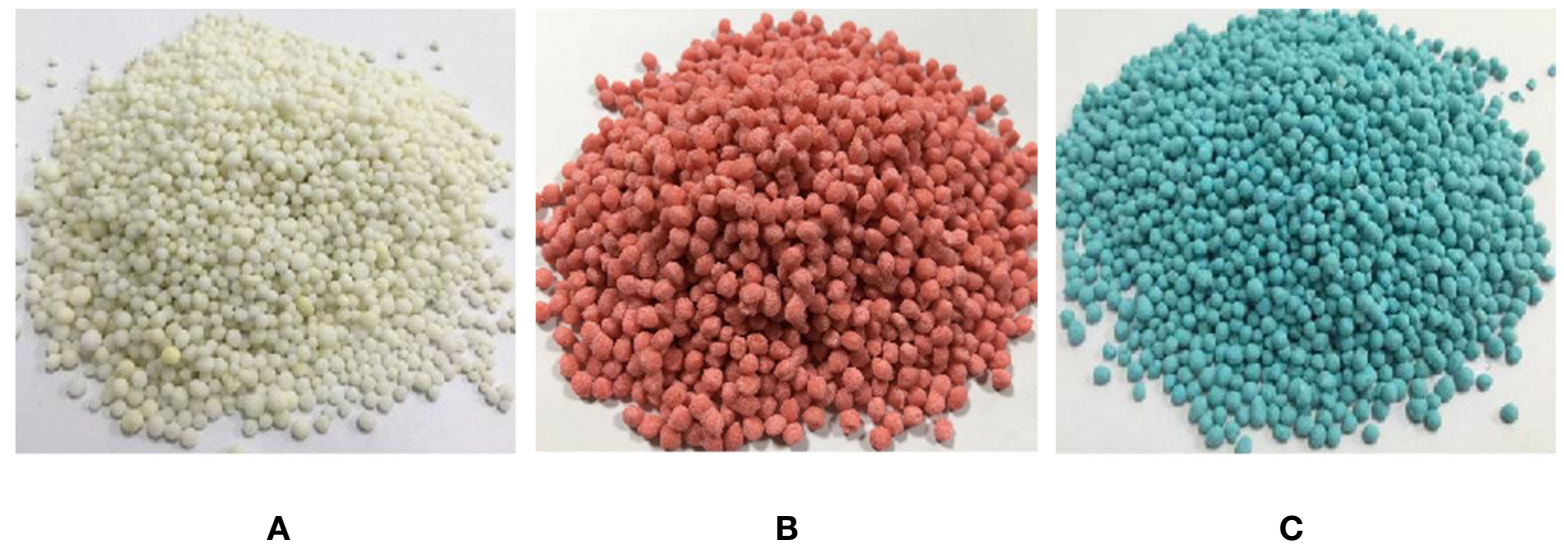
Three types of fertilisers commonly used in mango orchards: **(A)** urea, **(B)** potassium chloride, **(C)** compound fertiliser.

To verify the applicability of different fertilisers to the spiral fertiliser discharger optimized in this study, three types of fertilisers (urea, potassium chloride, and compound fertiliser) commonly used in mango orchards are taken as the research objects, as shown in **Figure 12**. The physical characteristic parameters of the three fertilisers are measured, and the results are presented in **Table 6**. Taking the mass flow rate and the variable coefficient of fertilizing stability as response indicators, the optimal parameter combination of the fertiliser discharger determined above was exploited to conduct different fertiliser applicability verification tests. Five groups of tests were carried out for each fertiliser, and each group of tests was repeated three times. The mass flow rate and the variable coefficient of fertilizing stability of the three fertilisers were calculated by formulas (26)-(28). Finally, the average relative error was calculated, and the test results were compared and analysed.

**Table 6:** The physical properties parameters of the three types of fertilisers.

Type	Average equivalent diameter (mm)	Spherical rate (%)	Density (g/cm^3^)	Water content (%)	Angle of repose (°)
Urea	3.581	91.08%	0.938	2.53%	33.7
Potassium chloride	3.463	87.41%	0.965	2.71%	35.9
Compound fertiliser	3.829	92.13%	0.913	3.28%	32.5

**Figure 13:**
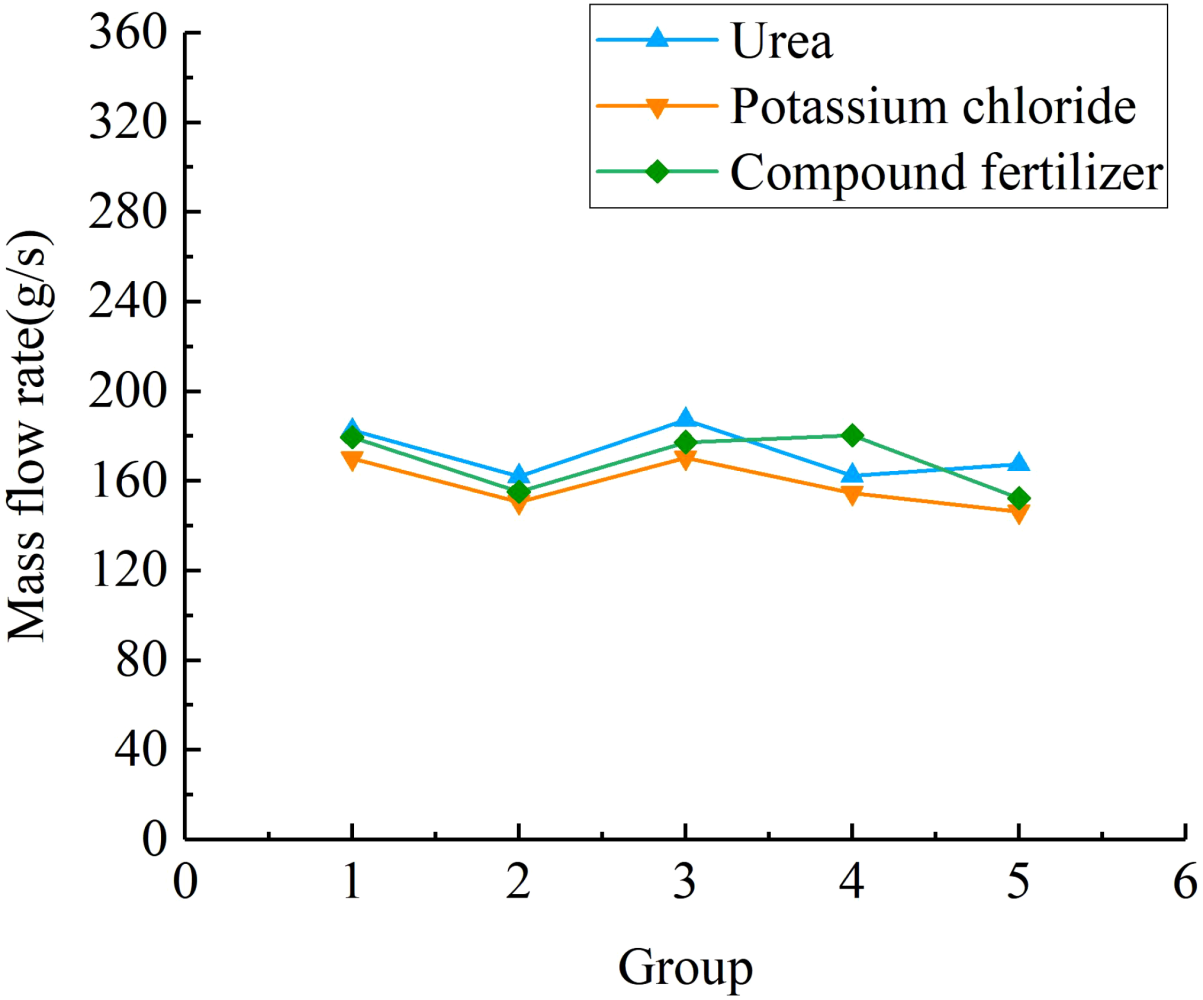
The comparison of mass flow rate results for different fertilisers.

**Figure 14:**
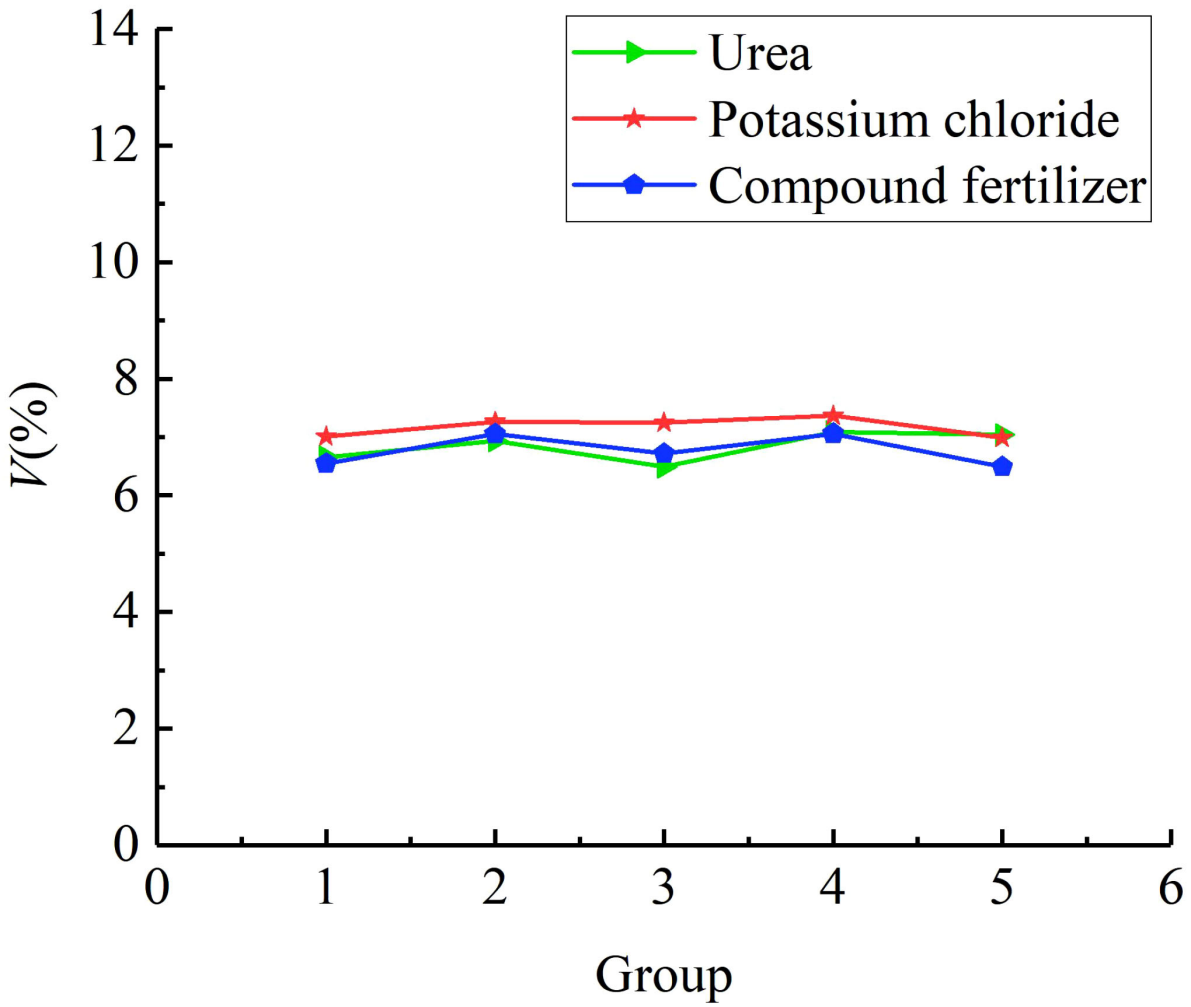
The comparison of fertilizing stability test results for different fertilisers.

**Figure 13** shows the comparison results of the fertiliser mass flow rate. According to statistical analysis, the average mass flow rate of urea particles, potassium chloride particles, and compound fertiliser particles is 172.35 g/s, 158.42 g/s, and 173.11 g/s, respectively. When the forward speed of the fertiliser applicator is 2 km/h and the plant spacing of mango trees is 1.82 m, the three types of fertilisers under the optimal parameter combination of the spiral fertiliser discharger can meet the fertilization requirements of 0.3-0.5 kg/plant for mango trees. **Figure 14** shows the comparison results of different fertilizing stability tests. The average variable coefficient of fertilizing stability of urea granules, potassium chloride granules, and compound fertiliser granules is 6.85%, 7.16%, and 6.73%, respectively. It can be seen that the average variable coefficient of fertilizing stability of the three types of fertilisers is small, which meets the agronomic fertilization requirements of the national standard “Technical Specification for Quality Evaluation of Fertilization Machinery” (the total variable coefficient of fertilizing stability ≤7.8% ). The applicability test results of different types of fertilisers further verified the reliability and accuracy of the discrete element simulation test results and the optimal parameter combination determined for the spiral fertiliser discharger. The designed small spiral fertiliser discharger can be used in the fertilization operation of mango orchards.

Through theoretical analysis, the error in the fluidity verification test of granular fertiliser may be due to the difference between the established discrete element model of granular fertiliser and the actual fertiliser. Through research, it is found that the average mass flow rate of potassium chloride particles in the applicability verification test of different fertilisers is smaller than that of urea and compound fertiliser particles, and the average variable coefficient of fertilizing stability of potassium chloride particles is larger than that of urea and compound fertiliser particles. It may be because the spherical rate of potassium chloride particles (87.41%) is smaller than that of urea (91.08%) and compound fertiliser (92.13%) particles, which leads to a slightly smaller mass flow rate of potassium chloride particles and a slightly larger coefficient of variation of fertiliser discharge stability. In addition, there are still some shortcomings in this study, such as considering only one moisture content, granular fertilisers with different moisture content may have different physical and mechanical properties and fertilizing performance, which will be further studied in the future. At the same time, the uniformity of fertiliser discharge is also an important index of the fertilizing performance of the spiral fertiliser discharger. The next step will be to build a relevant test platform and develop a mango orchard fertilization machine to conduct an experimental study on the uniformity of fertiliser discharge.

## 5 Conclusion

Based on the agronomic fertilization characteristics of mango orchards, a small spiral fertiliser discharger was designed in this study. First, the fertilizing performance test and parameter optimization of the spiral fertiliser discharger were conducted by combining the bench test and simulation test. Then, by taking the variable coefficient of fertilizing stability as the response value, the single-factor test and the quadratic regression orthogonal rotation combination design test were designed by the Design Expert software. Subsequently, the parameters significantly affecting the variable coefficient of fertilizing stability were explored, and the second-order regression mathematical model between the variable coefficient of fertilizing stability and the significant parameters was established. Finally, the optimal parameter combination of the fertiliser discharger was determined by the genetic algorithm. The accuracy of the simulation test results and the optimal parameter combination of the spiral fertiliser discharger was further verified by experiments. Based on the experimental results, the following conclusions were drawn:

(1) The single-factor test results showed that the diameter of the spiral blade, the pitch, and the rotational speed of the fertilizing shaft have significant effects on the variable coefficient of fertilizing stability, and the variable coefficient of fertilizing stability decreases with the increase in the diameter of the spiral blade and increases with the pitch and the rotational speed of the fertilizing shaft.
(2) The quadratic regression orthogonal rotation combination design test results showed that the established regression model of the variation coefficient of the fertiliser discharge stability has good reliability and precision. Meanwhile, the optimal parameter combination of the spiral fertiliser discharger optimized by the genetic algorithm is 98.44 mm for the diameter of the spiral blade, 54.8 mm for the pitch, and 24.43 r/min for the rotational speed of the fertilizing shaft. The variable coefficient of fertilizing stability is 6.51%, which meets the fertilization agronomic requirements of the national standard ‘Technical Specification for Quality Evaluation of Fertilization Machinery’.
(3) Under the optimal parameter combination of the fertiliser discharger, the verification test results on flowability and applicability of granular fertiliser show that the average relative error of the mass flow rate of the bench test and the simulated fertiliser discharge test is 2.64%, and the error is small. Meanwhile, the average mass flow rate of three types of fertilisers commonly used in mango orchards can meet the fertilization requirements of mango trees; the variable coefficient of fertilizing stability of the three types of fertilisers is small, which meets the agronomic fertilization requirements of mango orchards.

The above test results verify the reliability and accuracy of the discrete element simulation fertilizer test results and the optimal parameter combination of the determined fertilizer discharger. Therefore, the small spiral fertilizer discharger designed in this paper can be used for fertilization in mango orchards, which can effectively improve the accuracy of fertilization in mango orchards, the stability of fertilizer discharge and the utilization rate of fertilizer, thereby improving the yield and quality of mango, and playing a certain role in environmental protection. The results of this study provide basic data and research methods for the development of mango orchard fertilization machinery and other orchard related fertilizer performance tests.

## Data availability statement

The original contributions presented in the study are included in the article/**Supplementary Material**. Further inquiries can be directed to the corresponding authors.

## Author contributions

LZ: Methodology, Data curation, Software, Validation, Writing – original draft. HZ: Conceptualization, Supervision, Funding acquisition. LX: Writing – review & editing. WY: Formal analysis. MS: Visualization. JZ: Investigation. ZX: Resources. All authors contributed to the article and approved the submitted version.

## Glossary

DEM: discrete element method
GA: genetic algorithm
*Q*: the fertilizing amount
*D*: the diameter of the spiral blade
*n*: the rotational speed of the fertilizing shaft
*S*: the pitch
*v*: the forward speed of the fertilizer applicator
*f*: the fertilizer amount per mango tree
*R*: the average spacing length of mango trees
*K*_1_: the ratio coefficient between the diameter of the spiral blade and pitch
*J*: the fertiliser composite characteristic coefficient
*K*: the fertiliser synthesis factor
*m*: the mass of fertiliser
*r*: the radius of the spiral
*n_max_*: the critical speed of fertilizer shaft
*g*: the gravitational acceleration
*D*_1_: the equivalent diameter
*L*: the length
*W*: the width
*T*: the thickness
Φ: the spherical rate
*F_n_*: the normal force
*F_τ_*: the tangential force
$F_{d}^{n}$: the normal damping force
$F_{d}^{\tau }$: the tangential damping force
*E^*^*: the equivalent modulus of elasticity
*R^*^*: the equivalent radius
*E_i_*, *E_j_*: the modulus of elasticity
*v_i_*, *v_j_*: the poisson's ratio
*R_i_*, *R_j_*: the radius of contact particle
*S_τ_*: the tangential stiffness
*S_n_*: the normal stiffness
*G^*^*: the equivalent shear modulus
*m* ^*^: the equivalent mass
*E*: the recovery factor
$\nu_{n}^{\bar{rel}}$: the normal component of relative velocity
$\nu_{\tau }^{\bar{rel}}$: the tangential component relative velocity
*T_i_*: the moments
*Q_n_*: the fertilizing amount from a particular trial collected in the fertiliser monitoring area
*x*: the number of trials
*Y*: the total number of data points collected in the fertilizer monitoring area
*V*: the variable coefficient of fertilizing stability
*A*: the diameter of the spiral blade
*B*: the pitch
*C*: the rotational speed of fertilizing shaft
*AB*: the interaction of the diameter of the spiral blades and the pitch
*AC*: the interaction of the diameter of the spiral blades and the rotational speed of fertilizing shaft
*BC*: the interaction of the pitch and the rotational speed of fertilizing shaft
*A^2^*: the quadratic term of the diameter of the spiral blades
*B^2^*: the quadratic term of the pitch
*C^2^*: the quadratic term of the rotational speed of fertilizing shaft
*λ*: the fertilizer density
*ε*: the coefficient of inclined conveying
*ϕ*: the filling factor
*ω_max_*: the critical angular velocity of the fertilizer shaft
*δ_τ_*: the tangential overlap
*δ_n_*: the normal overlap
*β*: the damping ratio
*μ_s_*: the static friction factor
*μ_r_*: the rolling friction factor
*ω_i_*: the unit angular velocity vector of the granular fertiliser at the point of contact
*δ*: the average fertilizing amount for all data in the fertiliser monitoring area
*σ*: the standard deviation of the fertilizing amount for all data in the fertiliser monitoring area

## References

[B1] Bahiraei M. Nazari S. Moayedi H. Safarzadeh H. (2020). Using neural network optimized by imperialist competition method and genetic algorithm to predict water productivity of a nanofluid-based solar still equipped with thermoelectric modules. Powder Technol. 366, 571–586. doi: 10.1016/j.powtec.2020.02.055

[B2] Chen Z. Wang G. Xue D. Cui D. (2021). Simulation and optimization of crushing chamber of gyratory crusher based on the DEM and GA. Powder Technol. 384, 36–50. doi: 10.1016/j.powtec.2021.02.003

[B3] Coetzee C. J. Lombard S. G. (2011). Discrete element method modelling of a centrifugal fertiliser spreader. Biosyst. Eng. 109 (4), 308–325. doi: 10.1016/j.biosystemseng.2011.04.011

[B4] Dun G. Q. Liu W. H. Du J. X. Zhou C. Mao N. Ji W. Y. (2022). Optimization design and experiment of double spiral fertiliser discharger with arc groove. J. Agric. Machinery 53 (10), 118–125+174.

[B5] Dun G. Q. Liu Yang Y. Z. Chen H. T. Du J. X. Zhang J. T. (2018). Simulation and experiment of EDEM-based controlled-position layered fertiliser opener operation process. J. Hunan Agric. Univ. (Natural Sci. Edition) 44 (01), 95–100. doi: 10.13331/j.cnki.jhau.2018.01.018

[B6] He C. C. Feng H. D. Wei Z. Y. Hou X. W. Chen Y. Y. (2018). Investigation of current situation of fertilization and analysis of soil nutrients of mango orchard in HainanIsland. Chin. J. Trop. Crop 12, 2336–2348. doi: 10.3969/j.issn.1000-2561.2018.12.002

[B7] Huang S. Long D. He Y. (2018). Research progress and prospect of mango fertilization in china. Agric. Technol. 38 (16), 7–8. doi: 10.11974/nyyjs.20180833004

[B8] Jia H. L. Tan H. W. Wen X. Y. Wang G. Yuan H. F. Huang D. Y. (2022). Design and experiment of a pneumatic collection and discharge type precision blending and fertiliser application device. J. Agric. Machinery 53 (S2), 109–119+203. doi: 10.6041/j.issn.1000-1298.2022.S2.013

[B9] Liao Q. X. Chen Y. Zhang Q. S. Wang L. Ling J. X. Du W. B. (2023). Design and experiment of a side deep cavity fertiliser application device for oilseed rape. J. Agric. Machinery 54 (2), 1–13. [2022-12-06]. doi: 10.604/j.issn.1000-1298.2023.02.004

[B10] Liu J. S. Gao C. Q. Nie Y. J. Yang B. Ge R. Y. Xu Z. H. (2020). Numerical simulation of fertilizer shunt-plate with uniformity based on EDEM software. Comput. Electron. Agric. 178, 105737. doi: 10.1016/j.compag.2020.105737

[B11] Liu X. D. Ding Y. C. Shu C. X. Liu W. P. Wang K. Y. Du C. Q.. (2020). Design and experiment of spiral disturbance cone centrifugal fertilizer apparatus. Trans. CSAE 36 (2), 40–49. doi: 10.11975/j.issn.1002-6819.2020.02.006

[B12] Liu W. Si R. Fan J. Lin D. (2021). Effect of different fertilization patterns on yield and quality of mango. J. Trop. Crops 42 (03), 761–768. doi: 10.3969/j.issn.1000-2561.2021.03.022

[B13] Liu X. Wang X. Chen L. Zhang C. Liu W. Ding Y. (2021). Design and experiments of layered and quantitative fertilization device for rapeseed seeder [J]. Trans. CSAE 37 (5), 1–10. doi: 10.11975/j.issn.1002-6819.2021.05.001

[B14] Liu M. C. Zhao Q. J. Han S. Q. Song Z. H. Li F. D. Yan Y. F. (2022). Design and experiment of self-propelled variable ratio fertiliser directional spreader for mulberry gardens. J. Agric. Machinery 53 (S2), 120–130+140. doi: 10.6041/j.issn.1000-1298.2022.S2.014

[B15] Piri J. Mohapatra P. (2021). An analytical study of modified multi-objective harris hawk optimizer towards medical data feature selection. Comput. Biol. Med. 135, 104558. doi: 10.1016/j.compbiomed.2021.104558 34182329

[B16] Qu H. Peng J. Zhou L. Huang J. Zhu X. Zeng. Y. (2021). Analysis of fertiliser application status and reduction potential of orchards in baise mango main production area. J. South. Agric. 52 (12), 3375–3381. doi: 10.3969/j.issn.2095-1191.2021.12.021

[B17] Santana J. C. C. Araújo S. A. Alves W. A. Belan P. A. Jiangang L. Jianchu C.. (2018). Optimization of vacuum cooling treatment of postharvest broccoli using response surface methodology combined with genetic algorithm technique. Comput. Electron. Agric. 144, 209–215. doi: 10.1016/j.compag.2017.12.010

[B18] Sayed G. I. Soliman M. M. Hassanien A. E. (2021). A novel melanoma prediction model for imbalanced data using optimized SqueezeNet by bald eagle search optimization. Comput. Biol. Med. 136, 104712. doi: 10.1016/j.compbiomed.2021.104712 34388470

[B19] Song S. Duan J. Zou X. Yang Z. Ou Z. Wang B. (2020). Parameter optimization and test of variable fertilizer apparatus based on root distribution pattern of bananas. Trans. Chin. Soc Agric. Eng. 36, 11–18. doi: 10.11975/j.issn.1002-6819.2020.06.002

[B20] Sun J. Chen H. Duan J. Liu Z. Zhu Q. (2020). Mechanical properties of the grooved-wheel drilling particles under multivariate interaction influenced based on 3D printing and EDEM simulation. Comput. Electron. Agric. 172, 105329. doi: 10.1016/j.compag.2020.105329

[B21] Tan H. Xu L. Ma S. Niu C. Yan C. Shen C. (2022). Design and test of a scraper-type organic fertiliser strip spreading and rotary tillage hybrid fertiliser applicator. J. Agric. Machinery 1–24, 2022–12-06. doi: 10.6041/j.issn.1000-1298.2022.11.016

[B22] Thaper R. K. (2014) Effect of vane shape and fertilizer product on spread uniformity using a dual-disc spinner spreader (Doctoral dissertation, auburn university). Available at: https://www.proquest.com/dissertations-theses/effect-vane-shape-fertilizer-product-on-spread/docview/2778644784/se-2?accountid=44047.

[B23] Thawkar S. Sharma S. Khanna M. (2021). Breast cancer prediction using a hybrid method based on butterfly optimization algorithm and ant lion optimizer. Comput. Biol. Med. 139, 104968. doi: 10.1016/j.compbiomed.2021.104968 34735947

[B24] Van Liedekerke P. Tijskens E. Dintwa E. Rioual F. Vangeyte J. Ramon H. (2009). DEM simulations of the particle flow on a centrifugal fertilizer spreader. Powder Technol. 190 (3), 348–360. doi: 10.1016/j.powtec.2008.08.018

[B25] Wei P. (2021). How the largest mango base in china was built–baisai's observation of developing special industrial clusters based on resource advantages. Farmers' Friend 08), 2–7.

[B26] Wei Z. Aiping G. (2021). Research progress of mango nutrition and fertilization technology. China Forestry Specialties 02), 98–99+104. doi: 10.1016/j.powtec.2008.08.018

[B27] Wei Z. Y. Gao A. P. (2021). Current status and outlook of the application of discrete element method in agricultural engineering research. J. Agric. Machinery 52 (04), 1–20. doi: 10.13268/j.cnki.fbsic.2021.02.040

[B28] Xing J. Zhao H. Chen H. Deng R. Xiao L. (2023). Boosting whale optimizer with quasi-oppositional learning and gaussian barebone for feature selection and COVID-19 image segmentation. J. Bionic Eng. 20 (2), 797–818. doi: 10.1007/s42235-022-00297-8 36466725 PMC9707266

[B29] Yang L. Wang J. Sun X. Xu M. (2019). Multi-objective optimization design of spiral demister with punched holes by combining response surface method and genetic algorithm. Powder Technol. 355, 106–118. doi: 10.1016/j.powtec.2019.07.030

[B30] Yuan Q. Xu L. Ma S. Niu C. Yan C. Zhao S. (2021). The effect of paddle configurations on particle mixing in a soil-fertilizer continuous mixing device. Powder Technol. 391, 292–300. doi: 10.1016/j.powtec.2021.06.022

[B31] Zhao L. Zhou H. P. Xu L. Y. Song S. Y. Zhang C. Yu Q. X. (2022). Design and experiment of a powdered organic fertiliser strip application and discharge device. J. Agric. Machinery 53 (10), 98–107. doi: 10.1016/j.powtec.2021.09.065

[B32] Zhao L. Zhou H. Xu L. Song S. Zhang C. Yu Q. (2022). Parameter calibration of coconut bran substrate simulation model based on discrete element and response surface methodology. Powder Technol. 395, 183–194. doi: 10.1016/j.powtec.2021.09.065

[B33] Zhu X. H. Li X. D. Gao X. Tan C. Deng H. T. (2022). Design and test of a self-propelled organic fertiliser spreader for orchards. J. Agric. Machinery 53 (05), 136–146. doi: 10.6041/j.issn.1000-1298.2022.05.014

[B34] Zhu Q. Wu G. Chen L. Zhao C. Meng Z. (2018). Influences of structure parameters of straight flute wheel on fertilizing performance of fertilizer apparatus. Trans. CSAE 34 (18), 12–20. doi: 10.11975/j.issn.1002-6819.2018.18.002

