# Supplementary material for: Parameter optimization of the spiral fertiliser discharger for mango orchards based on the discrete element method and genetic algorithm
Source: Front Plant Sci. 2023 Nov 6;14:1169091. doi: 10.3389/fpls.2023.1169091 (PMC10952000; doi:10.3389/fpls.2023.1169091)
Supplement: Supplementary file 1 [file DataSheet_1.doc]

**The supplementary Figures**


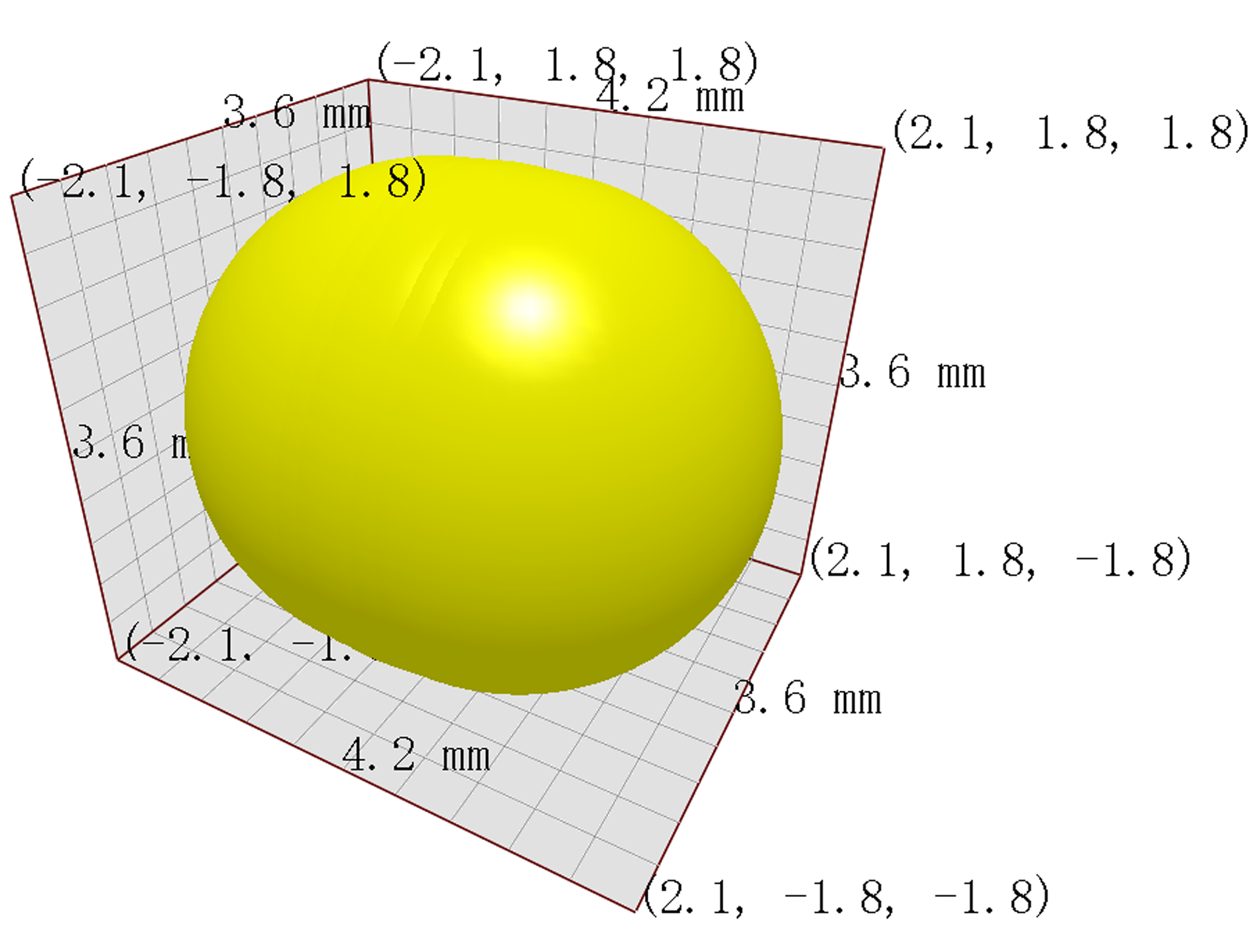


FIGURE 1 The discrete element model of the compound fertiliser particles





FIGURE 2 The curve of iterative convergence

**The supplementary Tables**

TABLE 1 The material comprehensive characteristics table.

| Material bulkiness | Abrasive properties | Object |  | $K$ | $J$ |
| --- | --- | --- | --- | --- | --- |
| Granular | abrasive | fertiliser | 0.20-0.35 | 0.0632 | 28 |
| Granular | Semi-abrasive | Maize | 0.25-0.30 | 0.4055 | 46 |
| Powder | Semi-abrasive | Lime | 0.30-0.40 | 0.0415 | 75 |
| Powder | Non-faceted | Flour | 0.25-0.35 | 0.0490 | 50 |
| Lumpy | Semi-abrasive | Ore | 0.15-0.20 | 0.0795 | 15 |

TABLE 2 The parameters related to the triaxial size of compound fertiliser particles

| Compound fertiliser | Length $L$ (mm) | Width $W$ (mm) | Thickness $T$(mm) | Equivalent diameter $D_{1}$ (mm) | Spheric ity $\Phi$ (%) |
| --- | --- | --- | --- | --- | --- |
| Mean | 4.168 | 3.819 | 3.535 | 3.829 | 92.13% |
| Standard deviation | 0.173 | 0.184 | 0.204 | 0.134 | 0.036 |

TABLE 3 The physical and mechanical parameters of the compound fertiliser particles

| Parameter | Poisson's ratio | Density (kg/m^3^) | Shear modulus (Mpa) |
| --- | --- | --- | --- |
| Q235 | 0.30 | 7810 | 4.5E+02 |
| Compound fertiliser | 0.25 | 913 | 1.0E+01 |
| Contact mechanics parameters | Compound fertiliser - compound fertiliser | collision recovery coefficient | 0.099 |
|  |  | Static friction coefficient | 0.662 |
|  |  | Rolling friction coefficient | 0.581 |
|  | Compound fertiliser - geometry | collision recovery coefficient | 0.381 |
|  |  | Static friction coefficient | 0.629 |
|  |  | Rolling friction coefficient | 0.554 |
