# Supplementary figures and images for: Parameter optimization of the spiral fertiliser discharger for mango orchards based on the discrete element method and genetic algorithm
Source: Front Plant Sci. 2023 Nov 6;14:1169091. doi: 10.3389/fpls.2023.1169091 (PMC10952000; doi:10.3389/fpls.2023.1169091)

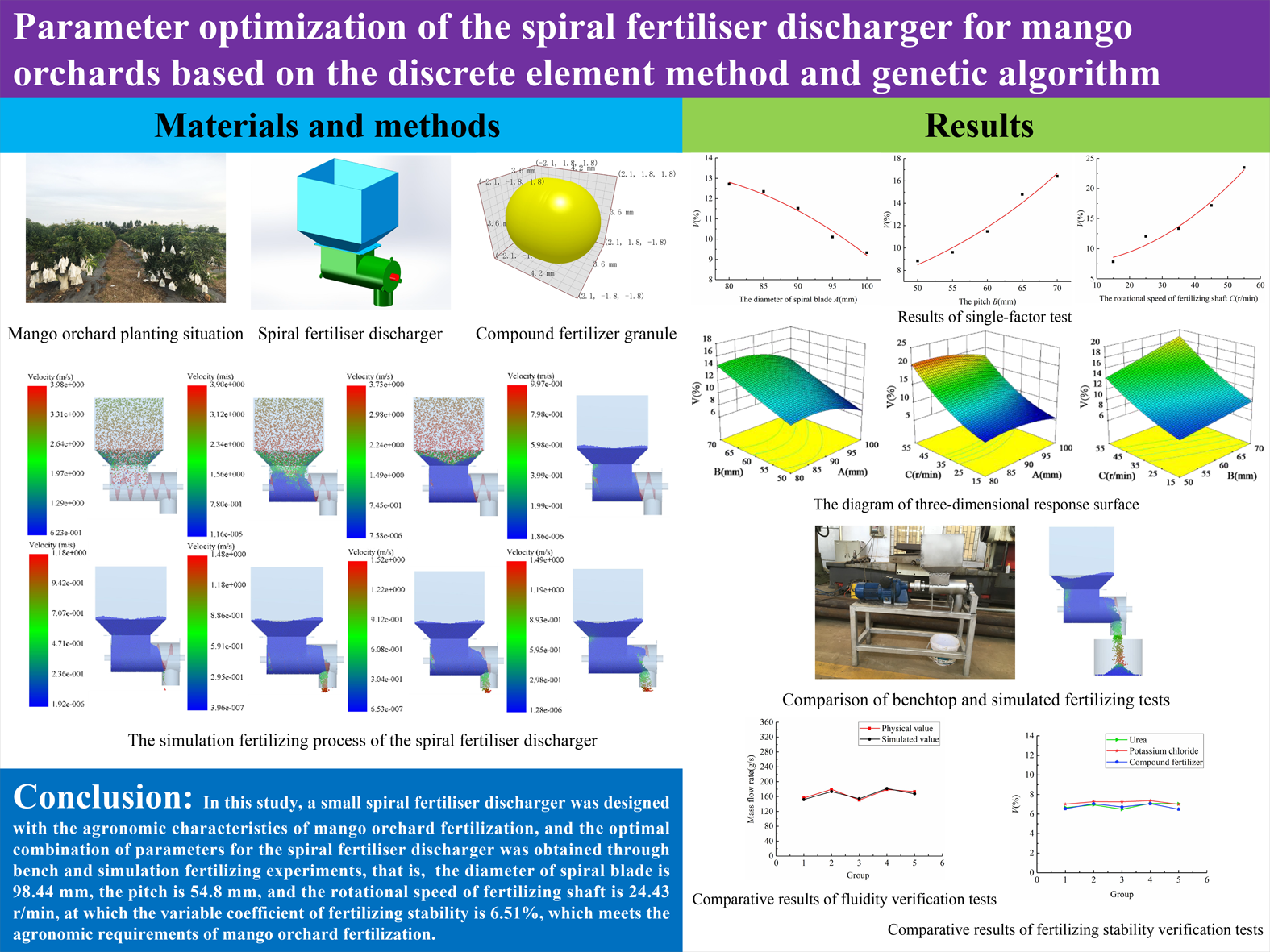

Supplement: Supplementary file 2 [file Image_1.tif]
